# VEGF-A/VEGFR-1 signalling and chemotherapy-induced neuropathic pain: therapeutic potential of a novel anti-VEGFR-1 monoclonal antibody

**DOI:** 10.1186/s13046-021-02127-x

**Published:** 2021-10-14

**Authors:** Laura Micheli, Carmen Parisio, Elena Lucarini, Alessia Vona, Alessandra Toti, Alessandra Pacini, Tommaso Mello, Serena Boccella, Flavia Ricciardi, Sabatino Maione, Grazia Graziani, Pedro Miguel Lacal, Paola Failli, Carla Ghelardini, Lorenzo Di Cesare Mannelli

**Affiliations:** 1 Department of Neuroscience, Psychology, Drug Research and Child Health - NEUROFARBA - Pharmacology and Toxicology Section, University of Florence, Viale G. Pieraccini 6, 50139 Florence, Italy; 2 Department of Experimental and Clinical Medicine - DMSC - Anatomy and Histology Section, University of Florence, L.go Brambilla 3, 50134 Florence, Italy; 3 Department of Biomedical, Experimental and Clinical Sciences, University of Florence, Viale G. Pieraccini 6, 50139 Florence, Italy; 4 Department of Experimental Medicine, Section of Pharmacology, University of Campania “L. Vanvitelli”, Via Santa Maria di Costantinopoli 16, 80138 Naples, Italy; 5 I.R.C.S.S., Neuromed, 86077 Pozzilli, Italy; 6 Department of Systems Medicine, University of Rome Tor Vergata, Via Montpellier 1, 00133 Rome, Italy; 7 IDI-IRCCS, Via Monti di Creta 104, 00167 Rome, Italy

**Keywords:** VEGF-A, VEGFR-1, Neuropathy biomarker, Astrocytes, D16F7 mAb, Oxaliplatin, Vincristine, Paclitaxel, Melanoma

## Abstract

**Background:**

Neuropathic pain is a clinically relevant adverse effect of several anticancer drugs that markedly impairs patients’ quality of life and frequently leads to dose reduction or therapy discontinuation. The poor knowledge about the mechanisms involved in neuropathy development and pain chronicization, and the lack of effective therapies, make treatment of chemotherapy-induced neuropathic pain an unmet medical need. In this context, the vascular endothelial growth factor A (VEGF-A) has emerged as a candidate neuropathy hallmark and its decrease has been related to pain relief. In the present study, we have investigated the role of VEGF-A and its receptors, VEGFR-1 and VEGFR-2, in pain signalling and in chemotherapy-induced neuropathy establishment as well as the therapeutic potential of receptor blockade in the management of pain.

**Methods:**

Behavioural and electrophysiological analyses were performed in an in vivo murine model, by using selective receptor agonists, blocking monoclonal antibodies or siRNA-mediated silencing of VEGF-A and VEGFRs. Expression of VEGF-A and VEGFR-1 in astrocytes and neurons was detected by immunofluorescence staining and confocal microscopy analysis.

**Results:**

In mice, the intrathecal infusion of VEGF-A (VEGF_165_ isoforms) induced a dose-dependent noxious hypersensitivity and this effect was mediated by VEGFR-1. Consistently, electrophysiological studies indicated that VEGF-A strongly stimulated the spinal nociceptive neurons activity through VEGFR-1. In the dorsal horn of the spinal cord of animals affected by oxaliplatin-induced neuropathy, VEGF-A expression was increased in astrocytes while VEGFR-1 was mainly detected in neurons, suggesting a VEGF-A/VEGFR-1-mediated astrocyte-neuron cross-talk in neuropathic pain pathophysiology. Accordingly, the selective knockdown of astrocytic VEGF-A by intraspinal injection of shRNAmir blocked the development of oxaliplatin-induced neuropathic hyperalgesia and allodynia. Interestingly, both intrathecal and systemic administration of the novel anti-VEGFR-1 monoclonal antibody D16F7, endowed with anti-angiogenic and antitumor properties, reverted oxaliplatin-induced neuropathic pain. Besides, D16F7 effectively relieved hypersensitivity induced by other neurotoxic chemotherapeutic agents, such as paclitaxel and vincristine.

**Conclusions:**

These data strongly support the role of the VEGF-A/VEGFR-1 system in mediating chemotherapy-induced neuropathic pain at the central nervous system level. Thus, treatment with the anti-VEGFR-1 mAb D16F7, besides exerting antitumor activity, might result in the additional advantage of attenuating neuropathic pain when combined with neurotoxic anticancer agents.

**Supplementary Information:**

The online version contains supplementary material available at 10.1186/s13046-021-02127-x.

## Background

The neurotoxic effects of anticancer chemotherapy induce a clinically relevant neuropathy (chemotherapy-induced neuropathy, CIN) that markedly impairs patients’ quality of life and frequently leads to dose reduction or therapy discontinuation. CIN continues after therapy withdrawal and beyond cancer resolution [1–3]; it is typically associated with neuropathic hypersensitivity deriving from a direct nervous tissue damage and the maladaptive response of both peripheral and central nervous system.

Studying the role of stem cells in relieving CIN, we recently highlighted an increase in plasma concentration of the vascular endothelial growth factor A (VEGF-A) in rats repeatedly administered with oxaliplatin. Stem cells were able to control pain and normalize VEGF-A suggesting the possible implication of this growth factor in neuropathy development [4]. However, the role of VEGF-A in pain signalling is debated as conflicting literature data suggest both algesic [5–8] and analgesic effects [9–13].

VEGF-A is an anti-parallel homodimeric protein that belongs to the “Cys-loop” superfamily of proteins. It is mainly known as a pro-angiogenic factor mediating blood vessel formation, vascular permeability, endothelial cell proliferation, differentiation, leakage, migration, survival, and motility [14]. Alternative splicing of the *Vegfa* gene selectively removes intron regions and joins specific combinations of exons to generate distinct VEGF-A isoforms, designated as VEGF_xxx,_ where xxx represents the number of amino acids present in the final protein sequence (i.e., VEGF_111_, VEGF_121_, VEGF_145_, VEGF_165_, which is the most commonly represented isoform, VEGF_189_, or VEGF_206_) [15]. A major site of alternative splicing occurs at exon 8, whereby proximal and distal splicing generates the VEGF_xxx_a (pro-angiogenic) and the VEGF_xxx_b (anti-angiogenic) isoforms, respectively [12, 15, 16]. Interestingly, in quiescent vessels the highest proportion of total VEGF-A is represented by VEGF_165_b [17]. Although the relevance of VEGF_165_b in physiopathological processes is controversial [18], several recent studies demonstrate the splicing mechanisms leading to VEGF_165_b generation [19] and its involvement in immunomodulation, retinopathies and cancer [20–22].

From an evolutionary perspective, VEGF-A emerged in the CNS of primitive organisms that lacked an established vasculature, suggesting a vessel-independent activity [23, 24]. Indeed, growing evidence indicates a diverse range of effects of VEGF-A on neural cells during development and in adulthood [23, 25]. In particular, it promotes CNS perfusion and induces direct neurotrophic effects in normal and pathological conditions and, as a permeability factor, VEGF-A modulates the blood-brain-barrier (BBB) functionality [26, 27].

The production of VEGF-A is mainly regulated by hypoxia via the hypoxia inducible factor and by several growth factors (including the epidermal and the platelet-derived growth factors), as well as by oncogenic mutations (*VHL*, *RAS*, *WNT*, *KRAS* signalling pathway genes) (reviewed in [28]). Cellular responses to VEGF-A are mainly driven by their cognate receptors, VEGFR-1-and-2, belonging to the class IV receptor tyrosine kinase family [29]. The well-known VEGFR-2 plays essential roles in physiological angiogenesis [30] and mediates the neuroprotective effects of VEGF-A [10, 31]. Conversely, VEGFR-1 has been associated with pathological processes such has inflammation and tumour-associated angiogenesis [32]. This receptor has a higher affinity for VEGF-A than VEGFR-2 and is widely expressed also in non-endothelial cells [15] (see [29] for a review about the physiological roles of VEGF-A mediated by its receptors).

We have recently generated an anti-VEGFR-1 mAb (D16F7) that has shown antitumor activity in orthotopic in vivo models of highly aggressive cancers such as melanoma and glioblastoma [33–35]. The VEGF-A receptor VEGFR-1 has been shown to be expressed in several components of the tumour microenvironment, besides tumour cells themselves: tumour infiltrating endothelial cells, and tumour-associated macrophages of the pro-tumour M2 phenotype, whose precursors are mobilized from the bone marrow and recruited to the tumour site through VEGFR-1 activation by specific ligands. In this context, blockade of VEGFR-1 by D16F7 results in: a) inhibition of tumour-associated angiogenesis; b) reduction of myeloid progenitor mobilization and tumour infiltration by M2 macrophages/microglia; c) increase the CD8/Tregs lymphocytes ratio within the tumour; d) inhibition of invasiveness and vasculogenic mimicry of VEGFR-1 positive tumour cells [33, 35, 36].

The present work dissects the pain modulatory properties of VEGF-A at the CNS level in physiological and neuropathic conditions using preclinical in vivo models of CIN. Moreover, the role of the different receptor subtypes in pain signalling and the impact of targeting the VEGF-A/VEGFRs system in pain relief were explored. Our findings indicate the direct involvement of VEGF-A/VEGFR-1 in mediating chemotherapy-induced neuropathic pain at the CNS level and the therapeutic potential of the anti-VEGFR-1 D16F7 mAb in attenuating this adverse effect.

## Methods

### Animals

Eight weeks old male CD-1 mice (Envigo, Varese, Italy) weighing 20–25 g at the beginning of the experimental procedure were used. Animals were housed in the “Centro Stabulazione Animali da Laboratorio” (University of Florence, Italy) and in “Stabulario Centralizzato di Ateneo” (University of Campania “Luigi Vanvitelli”, Naples, Italy) and used at least 1 week after their arrival. Mice were housed in cages measuring 26 cm × 41 cm; animals were fed with a standard laboratory diet and tap water ad libitum and kept at 23 ± 1 °C with a 12 h light/dark cycle (light at 7 am).

### Treatments

VEGF_165_b (cat. #3045-VE-025, R&D System, USA), PlGF-2 (cat. 465-PL/CF, R&D System, USA), VEGF-E (cat. #CYT-263, Prospec, Israel), D16F7 [33] and DC101 (catalogue #BE0060 BioCell, Boston, MA, USA) or vehicle (0.9% NaCl) were injected intrathecally (i.t.) in conscious mice at the indicated doses in 5 μl, as previously described [37]. Briefly, a 25-μl Hamilton syringe connected to a 30-gauge needle was intervertebrally inserted between the L4 and L5 region, and advanced 6 mm into the lumbar enlargement of the spinal cord. Behavioural measurements were performed before and 30 min, 1 h, 3 h and 6 h after the administration of compounds. DC101 or D16F7 were injected 15 min before the VEGFR-1/2 agonists when administered in the co-treatment experiments.

The scrambled siRNA or the specific VEGFR siRNA (VEGFR-1 or VEGFR-2 siRNA, Ambion Life Technologies, Monza, Italy) were i.t. injected twice spaced 24 h apart (3.3 μg/5 μl per mouse) at the lumbar level of the mice spinal cord. On the third day, behavioural measurements were conducted after administration of VEGFRs agonists. Mice were sacrificed between days 4th and 5th for western blot analysis. Target sequences of the anti-mouse VEGFRs siRNAs were as follows: VEGFR-1, sense strand 5′-GCAUCUAUAAGGCAGCGGAtt-3′ and antisense strand UCCGCUGCCUUAUAGAUGCtc-3′; VEGFR-2, sense strand 5′-CCCGUAUGCUUGUAAAGAAtt-3′ and antisense strand 5′-UUCUUUACAAGCAUACGGGct-3′.

### Adeno-associated virus (AAV) infection

A shRNAmir vector specific for mouse VEGF-A, labelled with green fluorescent protein (GFP) and containing a glial fibrillary acidic protein (GFAP) promoter (AAV1-GFAP-eGFP-mVEGFA-shRNAmir, 1.6 X 1013 GC/ml, Vector Biosystem Inc., Malvern, PA, USA), or a vector containing a scrambled version of VEGF-A were used. Mice were deeply anaesthetized by intraperitoneal (i.p.) injection of ketamine (100 mg kg^− 1^) (Ketavet, MSD Animal Health, Milan, Italy) and xylazine (10 mg kg^− 1^) (Rompum, 20 mg/ml, Bayer, Milan, Italy) and then were placed in a stereotaxic frame using the mouse spinal adaptor (Stoelting, Wood Dale, IL, USA). The skin was incised at Th12-L5 and the mouse muscles around the left side of the interspace between Th12-L1 and L4-L5 vertebrae were removed. The dura mater and the arachnoid membrane were then carefully incised using the tip of a 30G needle to allow vector infusion through a small window. Intraspinal injections were done using a 5-μl Hamilton syringe connected to a 34G needle. The needle was placed 0.5 mm lateral to the spinal midline at a depth of 0.4 mm from the dorsal surface of the spinal cord and 1 μl of VEGF-A shRNAmir or scrambled vectors was bilaterally injected at 0.25 μl/min with a digital microinjector (Stoelting). The needle was left on place for another 3 min to prevent backflow. The surgical site was then sutured with 3–0 silk and mice were kept on a heating pad until recovery.

### CIN in vivo models

In mice treated with oxaliplatin (Carbosynth, Pangbourne, UK; 2.4 mg kg^− 1^) the drug was administered i.p. for 2 weeks [38, 39]. Oxaliplatin was dissolved in a 5% glucose solution. Control animals received an equivalent volume of vehicle. Behavioural tests were performed on day 15 for the acute treatments. In mice injected spinally with the VEGF-A shRNAmir vector or with the corresponding scrambled vector, oxaliplatin was administered for 2 weeks (10 total injections), starting treatment 14  days after viral vector administration. Control animals received an equivalent volume of vehicle. Behavioural measurements were performed on days 3, 5, 9, 11, 13 and 15.

Mice treated with paclitaxel (Carbosynth; 2.0 mg kg^− 1^) were injected i.p. on four alternate days (days 1, 3, 5 and 8) [39, 40]. Paclitaxel was dissolved in a mixture of 10% saline solution and Cremophor EL, a derivative of castor oil and ethylene oxide that is clinically used as paclitaxel vehicle. Control animals received an equivalent volume of vehicle and behavioural measurements started on day 10.

Mice treated with vincristine (Carbosynth; 0.1 mg kg^− 1^) were injected i.p. for five consecutive days [41]. Vincristine was dissolved in saline solution and control animals received an equivalent volume of vehicle. Behavioural measurements started on day 8.

### Assessment of mechanical hyperalgesia (Paw pressure test)

Mechanical hyperalgesia was determined by measuring the latency in seconds to withdraw the paw away from a constant mechanical pressure exerted onto the dorsal surface [42, 43]. A 15 g calibrated glass cylindrical rod (10 mm diameter) chamfered to a conical point (3 mm diameter) was used to exert the mechanical force. The weight was suspended vertically between two rings attached to a stand and was free to move vertically. A single measure was made per animal and a cut-off time of 40 s was used.

### Assessment of thermal allodynia (Cold plate test)

Thermal allodynia was assessed using the Cold-plate test. With minimal animal-handler interaction, mice were taken from home-cages, and placed onto the surface of the cold-plate (Ugo Basile, Varese, Italy), maintained at a constant temperature of 4 °C ± 1 °C. Ambulation was restricted by a cylindrical Plexiglas chamber (diameter: 10 cm, height: 15 cm), with open top. A timer controlled by foot peddle began timing response latency from the moment the mouse was placed onto the cold-plate. Pain-related behaviour (licking of the hind paw) was observed, and the time (seconds) of the first sign was recorded. The cut-off time of the latency of paw lifting or licking was set at 30 s [44].

### Assessment of mechanical allodynia (von Frey test)

Mechanical allodynia was measured with the dynamic plantar aesthesiometer (von Frey instrument) (Ugo Basile), as described by Di Cesare Mannelli and colleagues [39] with minor modifications. Briefly, the mice were placed in individual Plexiglas cubicles (8.5 × 3.4 × 3.4 cm) on a wire mesh platform. After approximately 30 min accommodation period, during which exploratory and grooming activity ended, the mechanical paw withdrawal threshold was measured as the hind paw withdrawal responded to von Frey hair stimulation. The mechanical stimulus was delivered to the plantar surface of the mouse hind paw from below the floor of the test chamber by an automated testing device. A steel rod (2 mm) was pushed with electronic ascending force (0–5 g in 35 s). When the animal withdrew its hind paw, the mechanical stimulus was automatically withdrawn, and the force recorded to the nearest 0.1 g. Nociceptive response for mechanical sensitivity was expressed as mechanical withdrawal threshold in grams. The mean was calculated from six consecutive trials and averaged for each group of mice.

### Assessment of locomotor activity (Hole-board test)

The locomotor activity was evaluated by using the hole-board test. The apparatus consisted of a 40 cm square plane with 16 flush mounted cylindrical holes (3 cm diameter) distributed 4 × 4 in an equidistant, grid-like manner. Mice were placed on the centre of the board one by one and allowed to move about freely for a period of 5 min each. Two photobeams, crossing the plane from mid-point to mid-point of opposite sides, thus dividing the plane into 4 equal quadrants, automatically signalled the movement of the animal (counts in 5 min) on the surface of the plane (locomotor activity). Miniature photoelectric cells, in each of the 16 holes, recorded (counts in 5 min) the exploration of the holes (exploratory activity) by the mice [45].

### Electrophysiological recordings of nociceptive specific (NS) neurons

On the day of electrophysiological recordings, mice were initially anesthetized with tribromoethanol (Avertin, Winthrop laboratories, New York, NY, USA; 1.25%). After tracheal cannulation, a catheter was placed into the right external jugular vein, to allow continuous infusion of propofol (5–10 mg/kg/h, i.v.) and spinal cord segments L4-L6 were exposed by laminectomy, near the dorsal root entry zone, up to a depth of 1 mm [46]. An elliptic rubber ring (about 3 × 5 mm), sealed with silicone gel onto the surface of the cord, was used for topical spinal drug application and to gain access to spinal neurons. Animals were fixed in a stereotaxic apparatus (David Kopf Instruments, Tujunga, CA, USA) through clamps attached to the vertebral processes. Single unit extracellular activity of dorsal horn NS neurons was performed by using a glass-insulated tungsten filament electrode (3–5 MΩ) (FHC Frederick Haer & Co., ME, USA). Spinal neurons were defined as NS neurons, when they were responding only to high intensity (noxious) stimulation [47]. In particular, to confirm NS response patterns, each neuron was characterized by applying a mechanical stimulation to the ipsilateral hind paw using a von Frey filament with 97.8 mN bending force (noxious stimulation) for 2 s until it buckled slightly [48, 49]. Only neurons that specifically responded to noxious hind paw stimulation were considered for recordings. The recorded signals were visualized into a window discriminator, whose output was processed by an interface CED 1401 (Cambridge Electronic Design Ltd., UK) connected to iOS 5 PC. Spike2 software (CED, version 5) was used to create peristimulus rate histograms on-line and to store and analyse digital records of single unit activity off-line. The spontaneous and noxious-evoked neuronal activity was expressed as spikes/sec (Hz) and the effect of drugs was analysed as % variation of firing rate, frequency and duration of excitation. After recording a stable basal activity (15 min), topical spinal application of vehicle or drugs was performed, and each extracellular recording was monitored until 45–60 min post-injection. In particular, groups of animals were divided as follows: 1) VEGF_165_b (3 ng/5 μl, pro-nociceptive dose on NS neurons), 2) VEGF_165_b + DC101 (10 pg/5 μl, the highest non pro-nociceptive dose) and 3) VEGF_165_b + D16F7 (100 pg/5 μl). At the end of the experiment, animals were killed with a lethal dose of urethane.

### Western blot analysis

The lumbar spinal cord of mice was explanted and immediately frozen with liquid nitrogen. The frozen tissues were homogenized with lysis buffer containing 50 mM Tris-HCl pH 8.0, 150 mM NaCl, 1 mM EDTA, 0.5% Triton X-100 and complete protease inhibitors (Roche, Milan, Italy). The suspensions were sonicated on ice using three high intensity 10s bursts with a cooling period of 10s each burst and centrifuged at 13,000×g for 10 min at 4 °C. Protein concentrations were quantified by bicinchoninic acid test. Fifty μg of tissue homogenate were resolved with precast polyacrylamide gel (BOLT 4–12% Bis-Tris Plus gel; Thermo Fisher Scientific, Monza, Italy) before electrophoretic transfer to nitrocellulose membranes (Bio-Rad, Milan, Italy). The membranes were blocked with 1% BSA and 5% fat-free powdered milk in PBS containing 0.1% Tween 20 (PBST) and then probed overnight at 4 °C with primary antibodies specific for VEGFR-1, VEGFR-2, VEGF-A, GAPDH or α-tubulin (Table S1). The membranes were then incubated for 1 h in PBST containing the appropriate secondary anti-rabbit or anti-mouse antibody (Table S1). ECL (Enhanced Chemiluminescence Pierce, Rockford, IL, USA) was used to visualize peroxidase-coated bands. Densitometric analysis was performed using the ImageJ analysis software (ImageJ, NIH, Bethesda, MD, USA). Normalization for α-tubulin or GAPDH content was performed. The values were reported as percentages of controls arbitrarily set at 100%.

### Immunofluorescence staining and confocal imaging

After animal sacrifice, the L4-L5 segments of the spinal cord were exposed from the lumbovertebral column via laminectomy and identified by tracing the dorsal roots from their respective dorsal root ganglion. Formalin-fixed (and no-fixed, used for VEGFR-1 primary antibody) cryostat sections (7 μm) were washed 3x with phosphate-buffered saline (PBS) and then incubated, at room temperature for 1 h, in blocking solution (PBS, 0.3% Triton X-100, 5% albumin bovine serum; PBST). The sections were subsequently incubated with the anti-VEGFR-1, anti-VEGF-A, or anti-Aquaporin 4 (anti-AQP-4) (a marker of astrocytic endfeet) primary antibodies, overnight at 4 °C (Table S1). The following day, slides were washed 3× with PBS and then sections were incubated in the dark with goat anti-rabbit or anti-mouse IgG secondary antibodies labelled with Alexa Fluor 568, in PBST at room temperature for 2 h. After 3× PBS 0.3% Triton X-100 wash for 10 min, the sections were incubated with DAPI, as nuclear marker, at room temperature for 5 min and then the slides were mounted using Fluoromount™ (Life Technologies-Thermo scientific, Rockford, IL, USA).

For double immunofluorescence, on the first day, an anti-Iba-1 antibody was added and the slides were incubated overnight at 4 °C. Conversely, the sections to be labelled for GFAP or NeuN were incubated the second day for 2 h in the dark with mouse anti-GFAP Alexa Fluor 488-conjugated or mouse anti-NeuN Alexa Fluor 488-conjugated antibodies (Table S1). For triple immunofluorescence, on the first day, an anti-RECA-1 antibody was added and the slides incubated overnight at 4 °C; then, sections were incubated with the anti-mouse IgG labelled with Alexa Fluor 568 for 2 h. Thereafter, incubation with anti-VEGF-A and anti-GFAP antibodies was allowed overnight in the dark. Finally, anti-mouse IgG labelled with Alexa Fluor 488 and anti-rabbit IgG labelled with Alexa Fluor 647 were added for 2 h in the dark (Table S1).

Negative control sections (no exposure to the primary antisera) were processed concurrently with the other sections for all immunohistochemical studies. Images were acquired using a motorized Leica DM6000 B microscope equipped with a DFC350FX camera (Leica, Mannheim, Germany).

The colocalization area was calculated using the “colocalization” plugin of ImageJ (after evaluating the threshold value for each channel) and expressed as percentage relative to the value of the VEGFR-1 or VEGF-A area. The mean fluorescence intensity of VEGF-A, in control and oxaliplatin-treated animals, was calculated by subtracting the background (multiplied by the total area) from the VEGF-A integrated intensity. Analyses were performed on three different images for each animal, collected through a 20x objective.

For confocal analysis, images were acquired with a Leica SP2 AOBS confocal microscope using a sequential scan setting (exciting lasers 488 nm and 561 nm) to avoid channel bleed-through. Images were acquired though a 63 × 1.4 NA PL APO objective at voxel size of 232 nm (xy) and 121 nm (z).

Confocal images were processed and analysed using Fiji [50]. Briefly, images were deconvolved using Deconvolution Lab2 with a synthetic PSF and ICTM algorithm [51]. Colocalization analysis was performed with JACoP (Fiji plugin) [52] and manually set thresholds. Colocalization parameters were calculated from 8 confocal z-stacks for each analysis.

### Statistical analysis

Results were expressed as means ± SEM and the analysis of variance was performed by ANOVA test. A Bonferroni’s significant difference procedure was used as post-hoc comparison. *P* values less than 0.05 were considered significant. Data were analysed using “Origin® 10” software.

Electrophysiological data were analysed through one-way ANOVA followed by Dunnet’s multiple comparison post-hoc test for statistical significance within groups. Two-way ANOVA followed by Bonferroni post-hoc test, for comparison between groups, was calculated by using GraphPad Prism 7.0. The data and statistical analysis comply with the recommendations on experimental design and analysis in pharmacology [53]. For all experiments, data were collected by researchers blind to the treatments.

## Results

### Nociceptive effect of VEGFRs selective ligands infused intrathecally

**Fig. 1:**
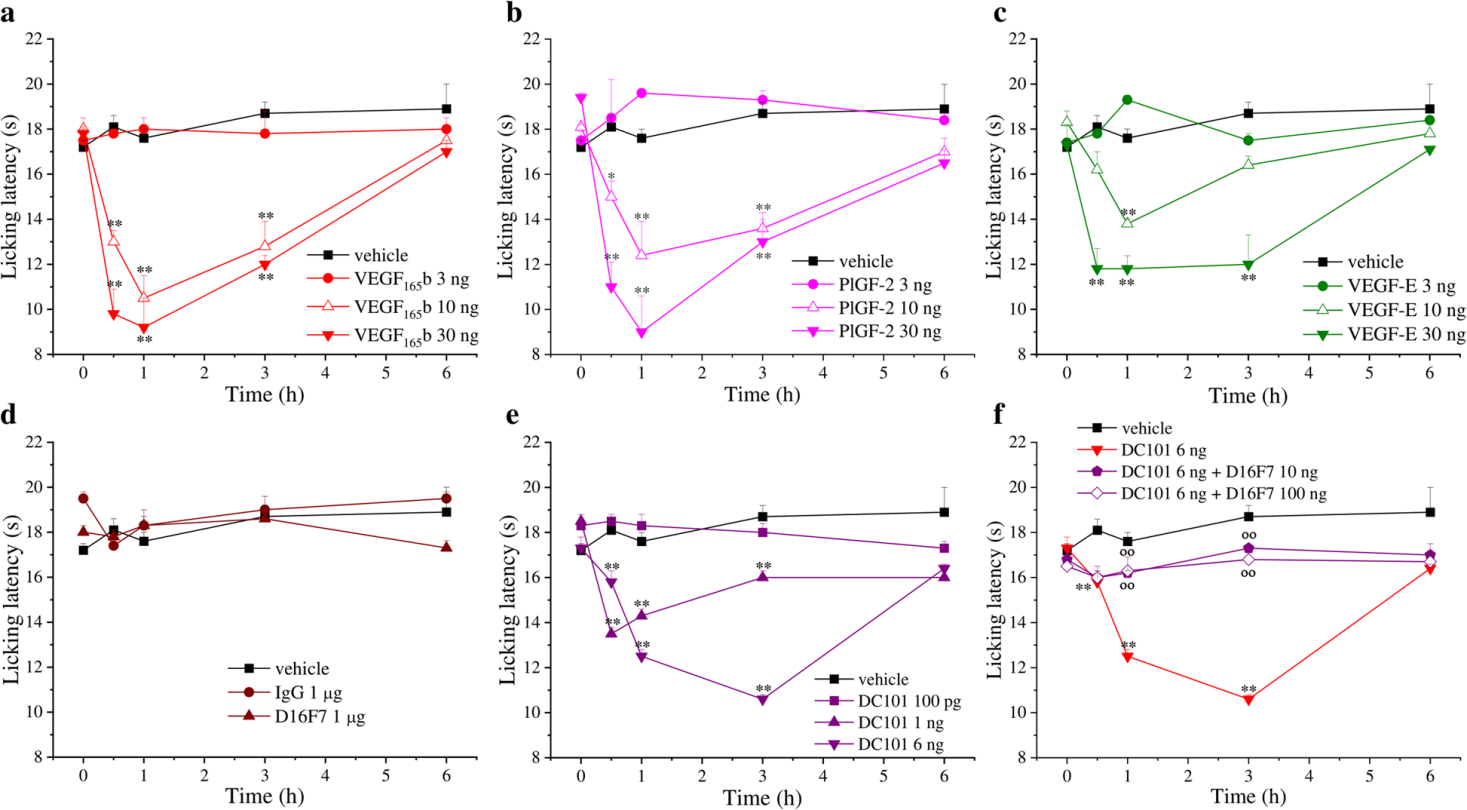
Nociceptive effect of VEGFRs selective ligands infused in spinal cord. The pain threshold was measured by the Cold plate test over time after the i.t. injection of the different molecules. Effect of (**a**) VEGF_165_b (*n* = 7), (**b**) the VEGFR-1 selective agonist PlGF-2 (*n* = 5), (**c**) the VEGFR-2 selective agonist VEGF-E (n = 5), (**d**) the selective anti-VEGFR-1 antibody D16F7 (n = 7) or a murine control IgG (n = 5) and (**e**) the selective anti-VEGFR-2 antibody DC101 (n = 5). (**f**) Effect of DC101 in mice pre-treated (15 min before) with D16F7. Each value represents the mean ± SEM. *P < 0.05 and **P < 0.01 vs vehicle-treated animals; °°P < 0.01 vs DC101 6 ng treated animals. The analysis of variance was performed by one-way ANOVA. A Bonferroni’s significant difference procedure was used as post-hoc comparison

To study the spinal impact of VEGF-A signalling modulation on pain threshold in mice, we firstly evaluated the effect of VEGF_165_b. This VEGF-A isoform was preferred since, differently from VEGF_165_a, it is able to specifically stimulate VEGFR-1 and VEGFR-2 without binding to VEGF-A co-receptors that may differentially interfere with the activation of the two tyrosine kinase receptors [54–56]. After i.t. administration of VEGF_165_b, pain sensitivity was measured as latency response to a cold stimulus (Cold plate test). As shown in Fig. 1a, VEGF_165_b (3, 10 and 30 ng, in bolus in a total volume of 5 μl) dose-dependently reduced pain threshold with a long-lasting effect starting 30 min after injection; this effect completely disappeared only after 6 h, similarly to what observed in rats [4]. Comparable dose-dependent nociceptive effects were observed with the other VEGF_165_ isoform (VEGF_165_a) (see Fig. S1). Since VEGF-A may interact with both VEGFR-1 and VEGFR-2, in order to explore the implications of the receptor types in pain modulation, we also tested the effect of placental growth factor 2 (PlGF-2) and VEGF-E, which are specific VEGFR-1 and VEGFR-2 agonists, respectively [57]. As shown in Fig. 1b and c, both PlGF-2 and VEGF-E (3, 10 and 30 ng, i.t.) significantly reduced the licking latency of animals challenged on a cold surface (Cold Plate test), even if PlGF-2 showed a profile similar to VEGF_165_b while VEGF-E exhibited a lower efficacy. Interestingly, the selective VEGFR-1 blockade by the anti-VEGFR-1 mAb D16F7 (injected i.t.), in the absence of VEGF_165_b, did not significantly alter pain threshold at microgram dose (Fig. 1d). On the contrary, nanogram dose of the anti-murine VEGFR-2 mAb DC101 (1 and 6 ng, i.t.) induced hypersensitivity (Fig. 1e) and this effect was blocked by D16F7 mAb (10 and 100 ng; Fig. 1f). In this test, non-specific mouse IgG (1 μg), used as control, was inactive. These findings suggested that the nociceptive effects evoked by VEGF_165_ isoforms were the result of VEGFR-1 stimulation. Furthermore, algesic effects induced by the DC101 mAb were likely due to the antibody-dependent displacement of the endogenous VEGF-A from VEGFR-2, thus making it available for binding to VEGFR-1; this hypothesis was further demonstrated by the loss of the effect when the anti-VEGFR-1 mAb D16F7, was administered together with DC101.

### Hypersensitivity-induced by VEGF-A signalling modulators is due to VEGFR-1 activation

**Fig. 2:**
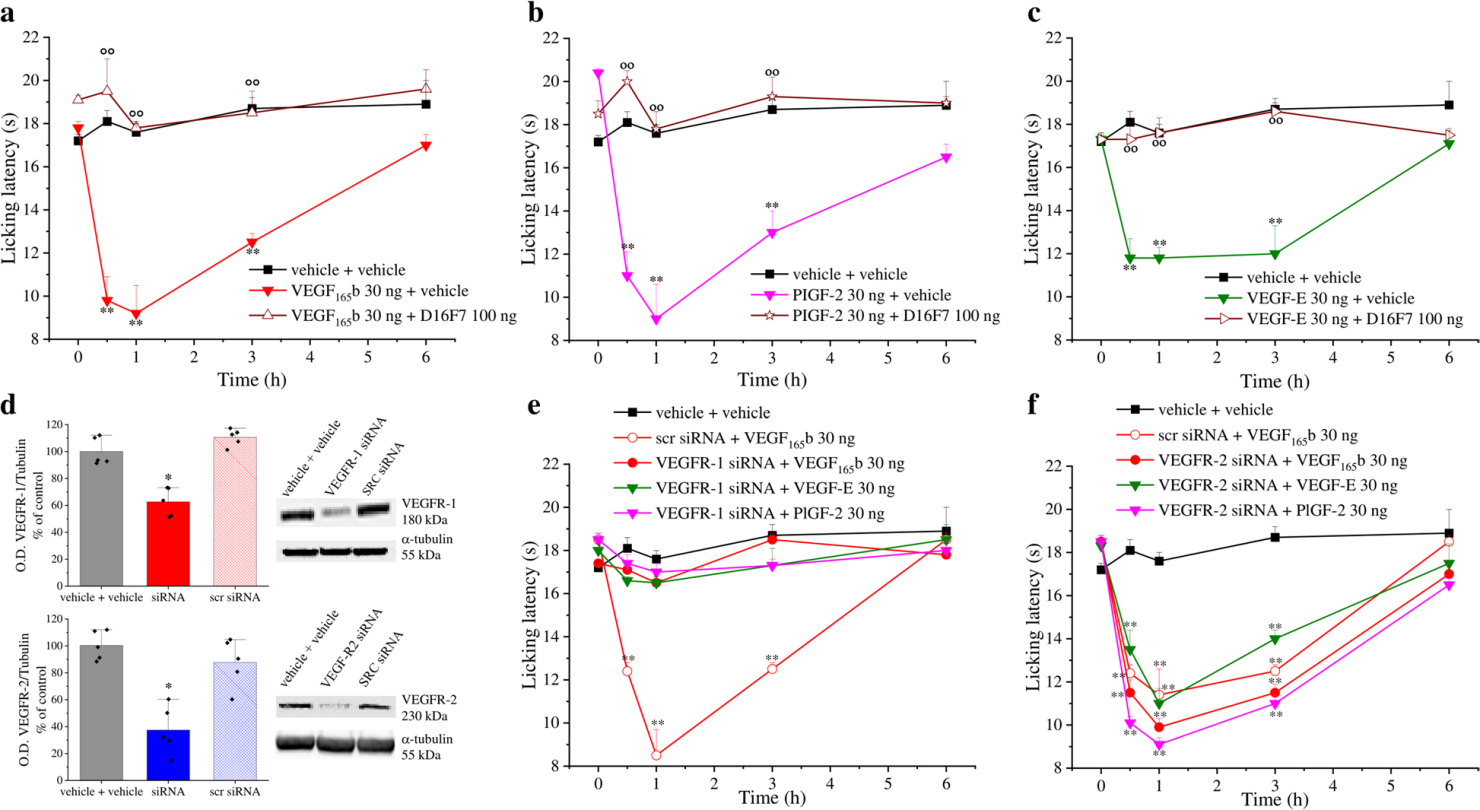
VEGF-A family members induce hypersensitivity through interaction with VEGFR-1. The response to a thermal stimulus (Cold plate test) was recorded after i.t. infusion of different VEGFR ligands (30 ng) preceded (15 min before) or not by the anti-VEGFR-1 mAb D16F7 (100 ng) or vehicle: (**a**) VEGF_165_b ± D16F7 (n = 5), (**b**) PlGF-2 ± D16F7 (n = 5), (**c**) VEGF-E ± D16F7 (n = 5). (**d**) Representative western blot images and densitometric analysis showing the expression of VEGFR-1 or VEGFR-2 in the lumbar section of the spinal cord after the siRNAs administration (*n* = 5). (**e** **-** **f**) Effects of VEGFR ligands (i.t.) in mice undergone a selective knockdown of VEGFR-1 (e, n = 5) or VEGFR-2 (f, n = 5) at the lumbar level of the spinal cord by siRNA. Each value represents the mean ± SEM. **P < 0.01 vs vehicle + vehicle-treated animals; °°P < 0.01 vs vehicle + VEGFRs ligands-treated animals. The analysis of variance was performed by one-way ANOVA. A Bonferroni’s significant difference procedure was used as post-hoc comparison

The hypothesis that VEGFR-1 activation is required for VEGF-A-mediated nociception was demonstrated by crossing the combinations of receptor agonists and antagonists. Both selective agonists, PlGF-2 and VEGF-E [58–61], share the same binding sites of VEGF-A on the corresponding receptors. At variance with DC101 mAb which is a competitive antagonist of VEGF-A and VEGF-E for VEGFR-2 binding [62], D16F7 mAb is a non-competitive antagonist since it interacts with VEGFR-1 at a site different from that used by the receptor ligands [32, 33]. Consistently with our hypothesis, the algesic effects of VEGF_165_b are blocked by D16F7 mAb (Fig. 2a). A similar profile was obtained also for the VEGFR-1 ligand PlGF-2 (Fig. 2b) as well as for the VEGFR-2 ligand VEGF-E (Fig. 2c). DC101 mAb used at the highest non-algesic dose (but able to selectively block VEGFR-2) [62] did not block the effect of both VEGF_165_b and PlGF-2, but further exacerbated VEGF-E hypersensitivity (Fig. S2). These findings confirmed the pivotal role of VEGFR-1 in pain signalling which is directly activated by the selective agonist PlGF-2 or by the exogenously added (Fig. 2a) or endogenously present VEGF-A (Fig. 2c) displaced from VEGFR-2. Moreover, the selective knockdown of VEGFR-1 or VEGFR-2 by siRNA further validated the specificity of the VEGFR-1-mediated mechanism (Fig. 2d-f). Silencing of VEGFR-1 completely blocked the effects of VEGF_165_b, PlGF-2 and VEGF-E (Fig. 2e), whereas silencing of VEGFR-2 did not alter the algesic properties of these ligands (Fig. 2f).

### VEGF_165_b increases the activity of spinal NS neurons by VEGFR-1 activation

**Fig. 3:**
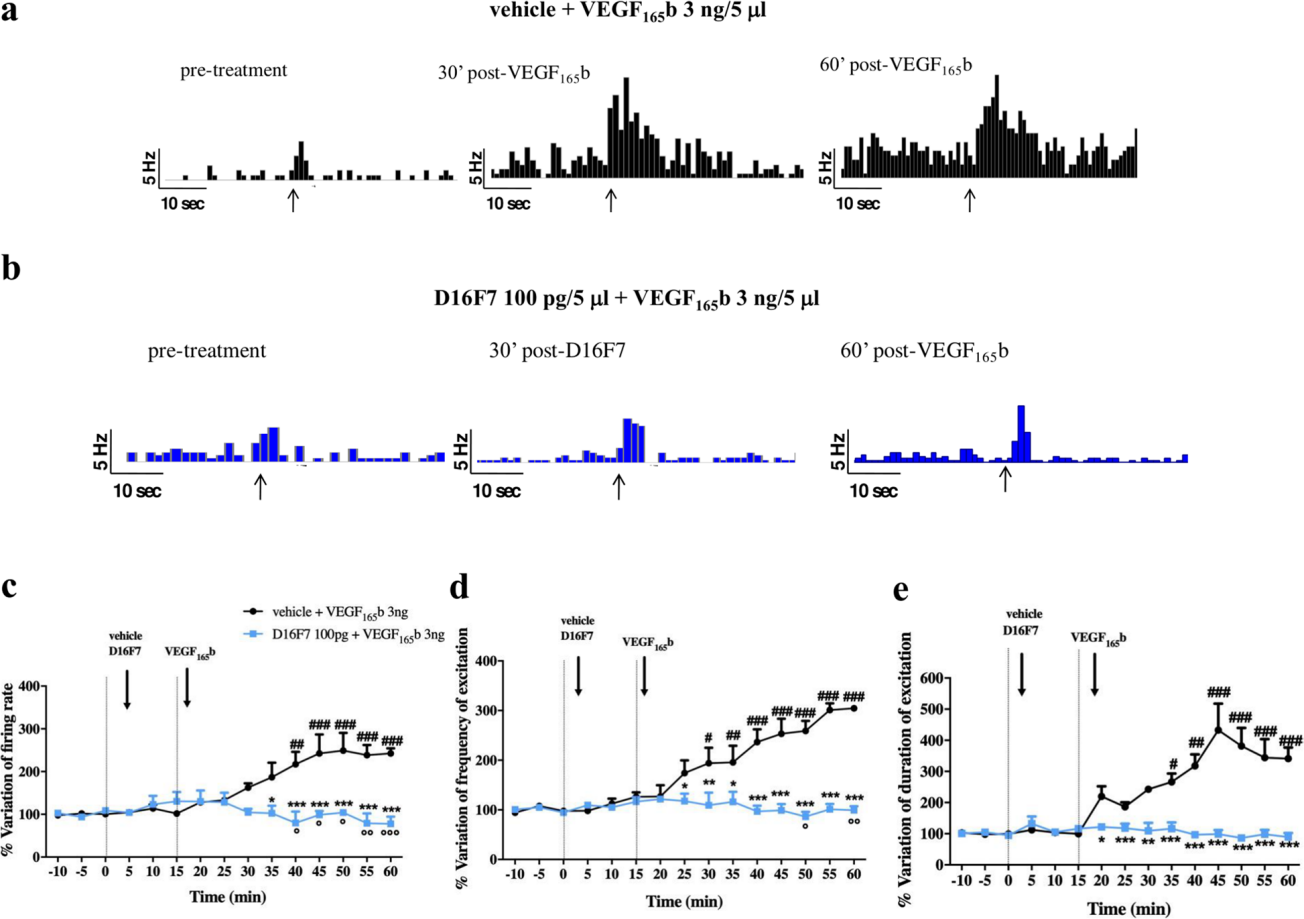
VEGF_165_b increases spontaneous and noxious-evoked activity of NS neurons through VEGFR-1. Representative ratematers showing spontaneous and noxious-evoked activity of NS neurons after spinal application of VEGF_165_b alone or in combination with D16F7 mAb (**a** and **b**, respectively); black arrows indicate the noxious stimulation on the mouse hind-paw. Mean ± SEM population data of spinal cord application of VEGF_165_b (3 ng/5 μl) in the presence of vehicle (0.9% NaCl), or D16F7 (100 pg/5 μl) on firing rate of spontaneous activity (**c**), frequency (**d**) and duration of evoked activity (**e**) of NS neurons in CD1 mice. Black arrows indicate vehicle, D16F7 or VEGF_165_b spinal application. Each point represents the mean of 5 different mice per group (one neuron recorded per each mouse). ^#^*P* < 0.05, ^##^*P* < 0.01 and ^###^*P* < 0.001 indicate statistical difference vs baseline; *P < 0.05, **P < 0.01 and ***P < 0.001 indicate statistical difference vs vehicle + VEGF_165_b. One-way ANOVA followed by Dunnet’s multiple comparison post-hoc test was performed for statistical significance within groups. Two-way ANOVA followed by Bonferroni post-hoc test was used for comparison between groups

To investigate the effect of the spinal application of VEGF_165_b on the hyperexcitability of spinal NS neurons, in vivo electrophysiological experiments were performed. The results relate to NS neurons (one cell recorded from each animal per treatment) localized at a depth of 0.7–1.0 mm from the surface of the spinal cord. This cell population was characterized by a mean rate of basal firing of 0.015 ± 0.002 spikes/sec and only cells showing this pattern were chosen for the experiment. To investigate the involvement of the different VEGF-A receptor subtypes, spontaneous and noxious-evoked (mechanical stimulation) activity of NS neurons was measured after spinal application of VEGF_165_b, preceded or not by treatment with the anti-VEGFR-1 mAb D16F7. Representative ratematers of the results obtained with VEGF_165_b in the absence or presence of D16F7 mAb are shown in Fig. 3a and b, respectively. In mice pre-treated with vehicle (0.9% NaCl), VEGF_165_b (3 ng/5 μl) spinal application induced an increase in spinal electrical activity as compared to baseline levels (100%). In particular, NS neurons showed a variation of spontaneous activity compared to baseline of 217.05 ± 29.2% as well as a noxious-evoked activity with frequency of 234 ± 30.9% and duration of 316.2 ± 27.2%, starting from 25 min post VEGF_165_b (Fig. 3a, c-e). The spinal VEGF_165_b-induced hypersensitivity was mainly mediated by VEGFR-1 rather than VEGFR-2 activation. Indeed, electrophysiological recordings revealed that spinal pre-application of D16F7 mAb (100 pg/5 μl) significantly prevented the increase of spontaneous and noxious-induced activity of NS neurons resulting in a pattern similar to baseline (Fig. 3b, c-e). D16F7 (100 pg/5 μl) alone was not able to affect either spontaneous or evoked activity of NS neurons (Fig. 3b). On the contrary, DC101 at 30 and 100 pg, showed a pro-nociceptive effect on spinal NS neuron activity per se (Fig. S3, representative ratematers). In fact, post-injection level of either spontaneous (187.3 ± 17.7% at 100 pg and 151.1 ± 6.9% at 30 pg) or noxious pressure-evoked firing rates (frequency: 212.6 ± 27% at 100 pg and 152.9 ± 6.9% at 30 pg; duration: 235.7 ± 25.3% at 100 pg and 119.7 ± 8.6% at 30 pg) were significantly higher respect to the baseline, in a dose-dependent manner. Overall, these results further confirmed the involvement of VEGFR-1 in VEGF-A-induced electrophysiological changes of NS neurons.

### VEGF-A and VEGFR-1 localization in the spinal cord of naïve mice

**Fig. 4:**
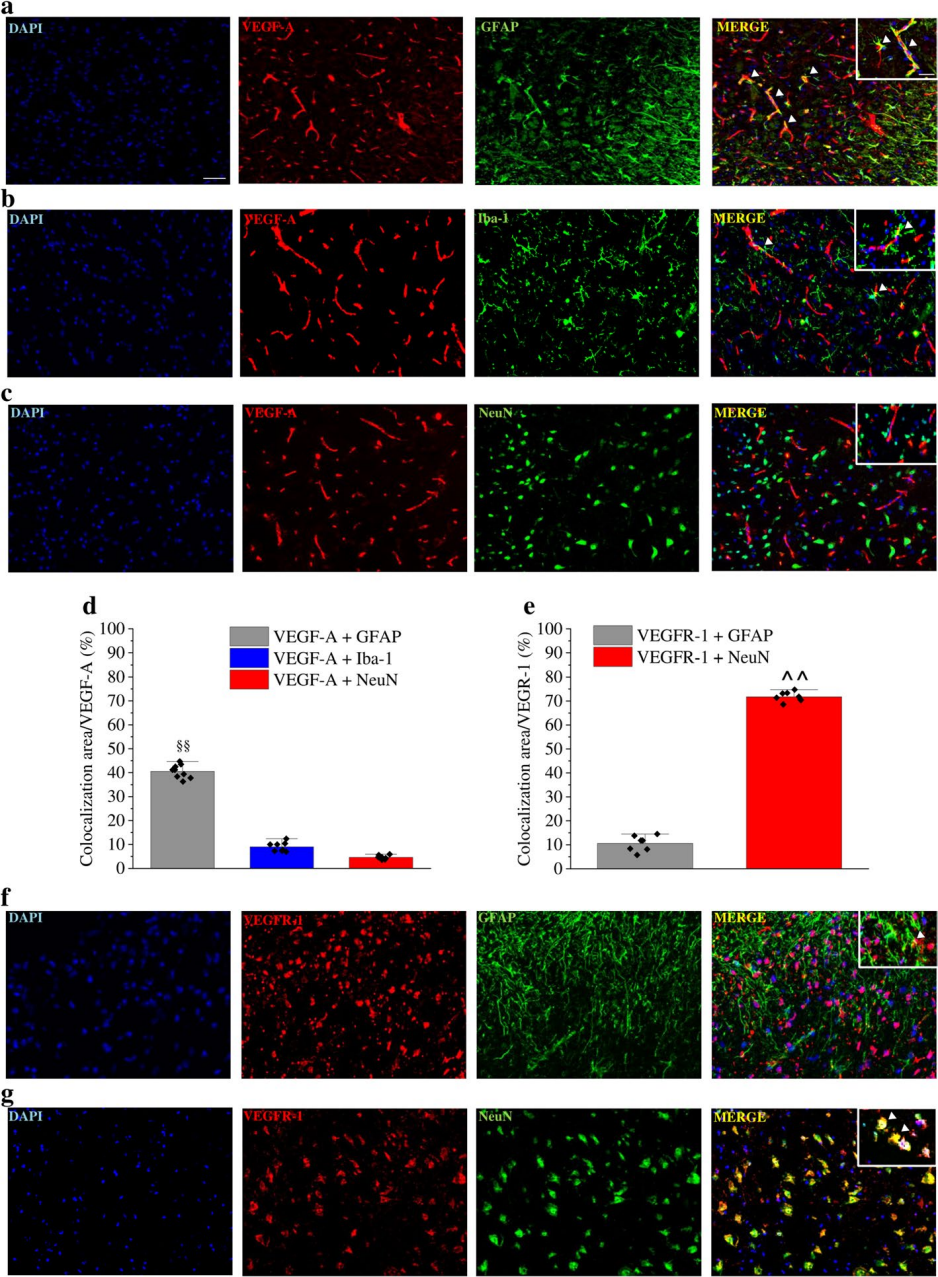
Cellular localization of VEGF-A and VEGFR-1 in the spinal cord of naïve mice. VEGF-A immunoreactivity was analysed in the spinal cord dorsal horn of naïve mice. Colocalization with GFAP-positive astrocytes (**a**, *n* = 9), Iba-1 positive microglia (**b**, *n* = 8) and NeuN-positive neurons (**c**, n = 7) was evaluated and quantified (**d**). Quantitative analysis (**e**) of immunofluorescence co-staining of VEGFR-1 in the dorsal horn with GFAP (**f**, n = 7) or NeuN (**g**, n = 8) positive cells. Scale bar: 100 μm. Each value represents the mean ± SEM. ^§§^P < 0.01 vs VEGF-A + Iba-1 and VEGF-A + NeuN. ^^P < 0.01 vs VEGFR-1 + GFAP. ﻿The analysis of variance was performed by Oone-way ANOVA. A Bonferroni’s significant difference procedure was used as post-hoc comparison﻿

**Fig. 5:**
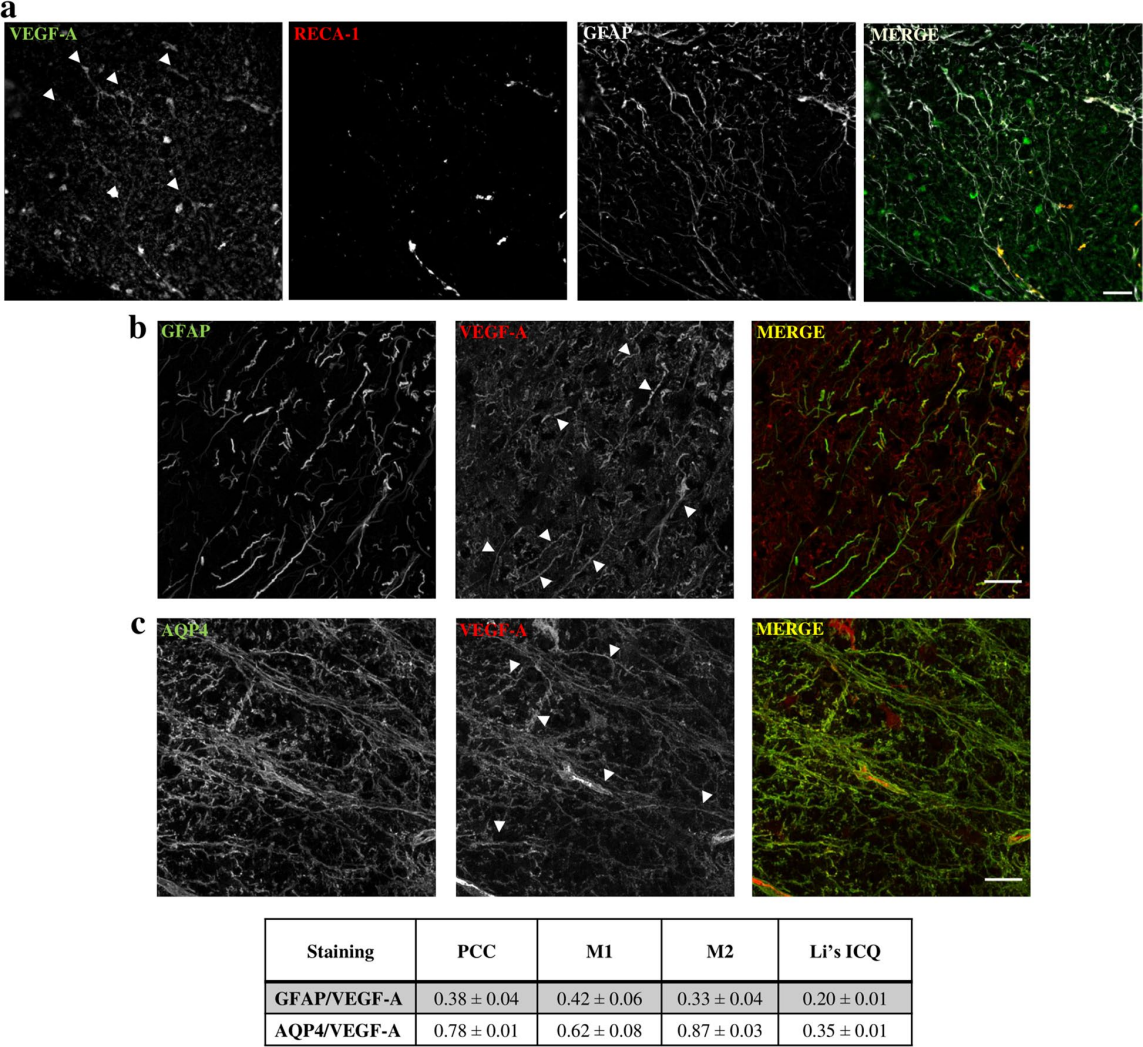
VEGF-A expression in endothelial and astrocytic cells of murine spinal cord. (**a**) VEGF-A immunoreactivity was analysed in the spinal cord dorsal horn of naïve mice in comparison to RECA-1-positive endothelial cells and GFAP-positive astrocytes; arrows indicate the presence of VEGF-A in astrocytes; scale bar: 100 μm. (**b** and **c**) Deconvolved confocal z-stacks shown as maximum intensity projection. Arrows indicate points of interest. (**b**) Representative GFAP and VEGF-A z-stack. (**c**) Representative VEGF-A and aquaporin-4 z-stack. Table). Colocalization parameters are given as mean ± SEM (n = 8), PCC = Pearson’s Correlation Coefficient; M1 = Mander’s M1; M2 = Mander’s M2; Li′s ICQ = Li′s Intensity Correlation Quotient. Colocalization graphs are shown in Fig. S4

Immunofluorescence analysis was performed in the dorsal horn of the spinal cord to study VEGF-A and VEGFR-1 expression profile in the nerve cells. VEGF-A immunoreactivity in astrocytes (as colocalization with GFAP; Fig. 4a and d) was significantly higher in comparison to microglia (Iba-1 positive cells) and neurons (NeuN positive cells) (Fig. 4b, c and d). As expected, VEGF-A staining was strictly related to vessel structure (Fig. 4a, c and d) since its expression was observed both on endothelial cells and astrocyte endfeet [25]. To better investigate this aspect, we compared the co-localization of VEGF-A with GFAP and RECA-1, a marker of endothelial cells (Fig. 5 and Fig. S4). As shown in Fig. 5a, it is possible to identify separate areas of VEGF-A/GFAP and VEGF-A/RECA-1 colocalization. Furthermore, VEGF-A expression in astrocytes was also confirmed by confocal microscopy. Results shown in Fig. 5b and c confirm the colocalization of VEGF-A with GFAP and AQP4. Indeed, the Van Steensel’s cross-correlation function (CCF) clearly shows that VEGF-A co-localizes with GFAP and AQP4 in cellular structures with estimated diameters of 1.00 ± 0.11 μm and 1.28 ± 0.12 μm (CCF at FWHM, mean ± SD, Fig. S4), respectively, which are compatible with the size of astrocytic processes. Collectively, these analyses demonstrate the presence of a VEGF-A pool in astrocytes.

VEGFR-1 expression was, instead, more prominent in neurons than in astrocytes (Fig. 4e, f and g).

### VEGF-A is increased in spinal astrocytes of mice with oxaliplatin-induced neuropathy

**Fig. 6:**
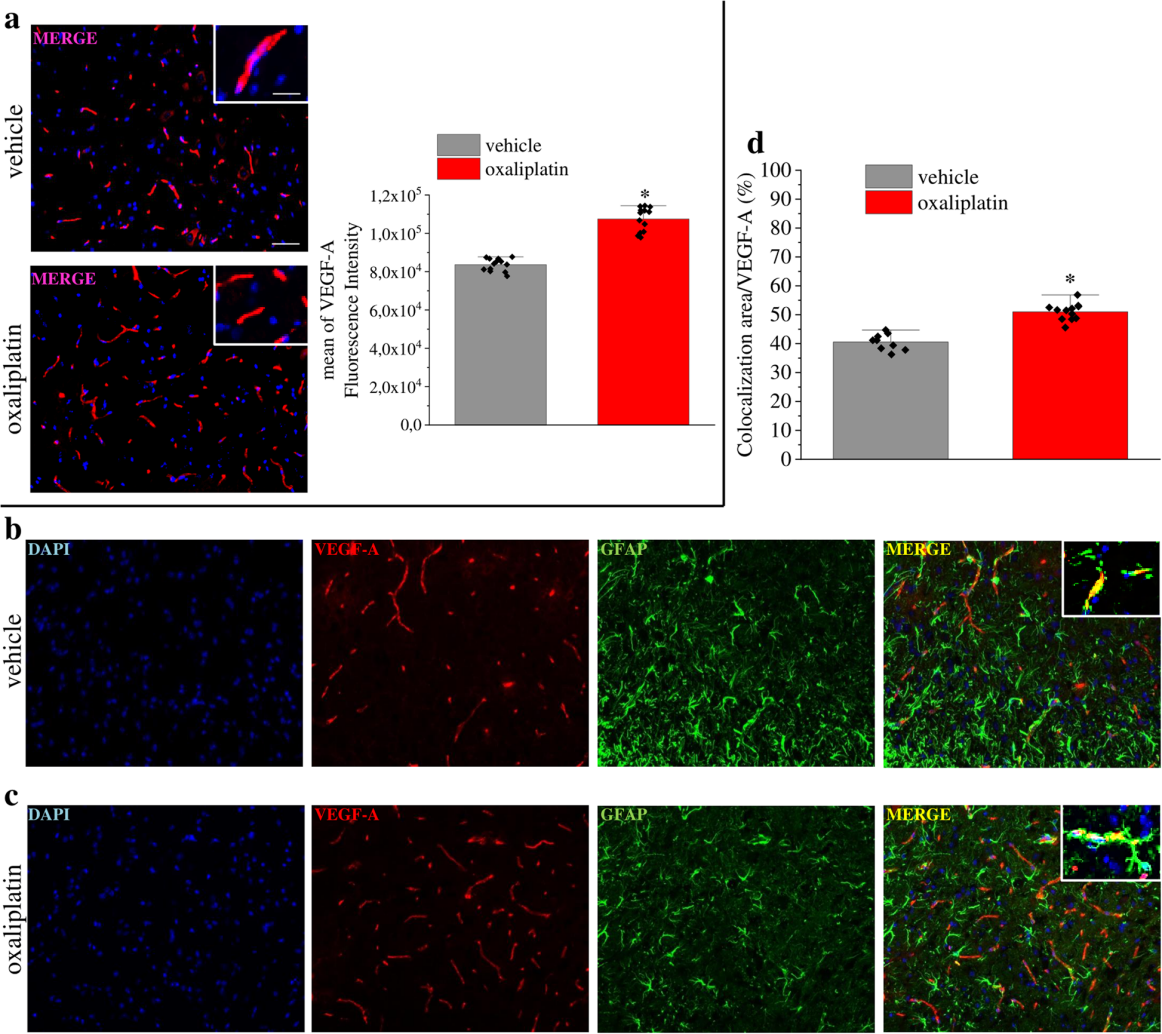
VEGF-A is increased in spinal astrocytes of mice with oxaliplatin-induced neuropathy. (**a**) Representative images and quantitative analysis of mean VEGF-A fluorescence intensity in the dorsal horn of oxaliplatin-treated mice in comparison to control animals (vehicle, *n* = 13). (**b**-**d**) Colocalization analysis of VEGF-A and GFAP in control (**b**) and oxaliplatin-treated mice (**c**). Quantitative analysis of colocalization area (**d**) (vehicle, n = 13; oxaliplatin, *n* = 12). Scale bar: 100 μm; insert: 50 μm. Each value represents the mean ± SEM. *P < 0.05 vs vehicle group. The analysis of variance was performed by one-way ANOVA. A Bonferroni’s significant difference procedure was used as post-hoc comparison

A painful neuropathy was reproduced in mice by a repeated treatment with oxaliplatin [38, 39]. After 2 weeks of treatment, when hypersensitivity was developed, VEGF-A immunoreactivity significantly increased in dorsal horns of the spinal cord in comparison to control animals (Fig. 6a and Fig. S5). The increment was specifically confirmed in astrocytes when colocalization of VEGF-A expression in GFAP-positive cells was measured (Fig. 6b, c and d). In regard to VEGFRs, VEGFR-2 expression increased in the spinal cord of oxaliplatin-treated mice, as revealed by western blot; on the contrary VEGFR-1 was unaffected by treatment with chemotherapy (Fig. S6).

### VEGF-A silencing in astrocytes prevents neuropathic pain

**Fig. 7:**
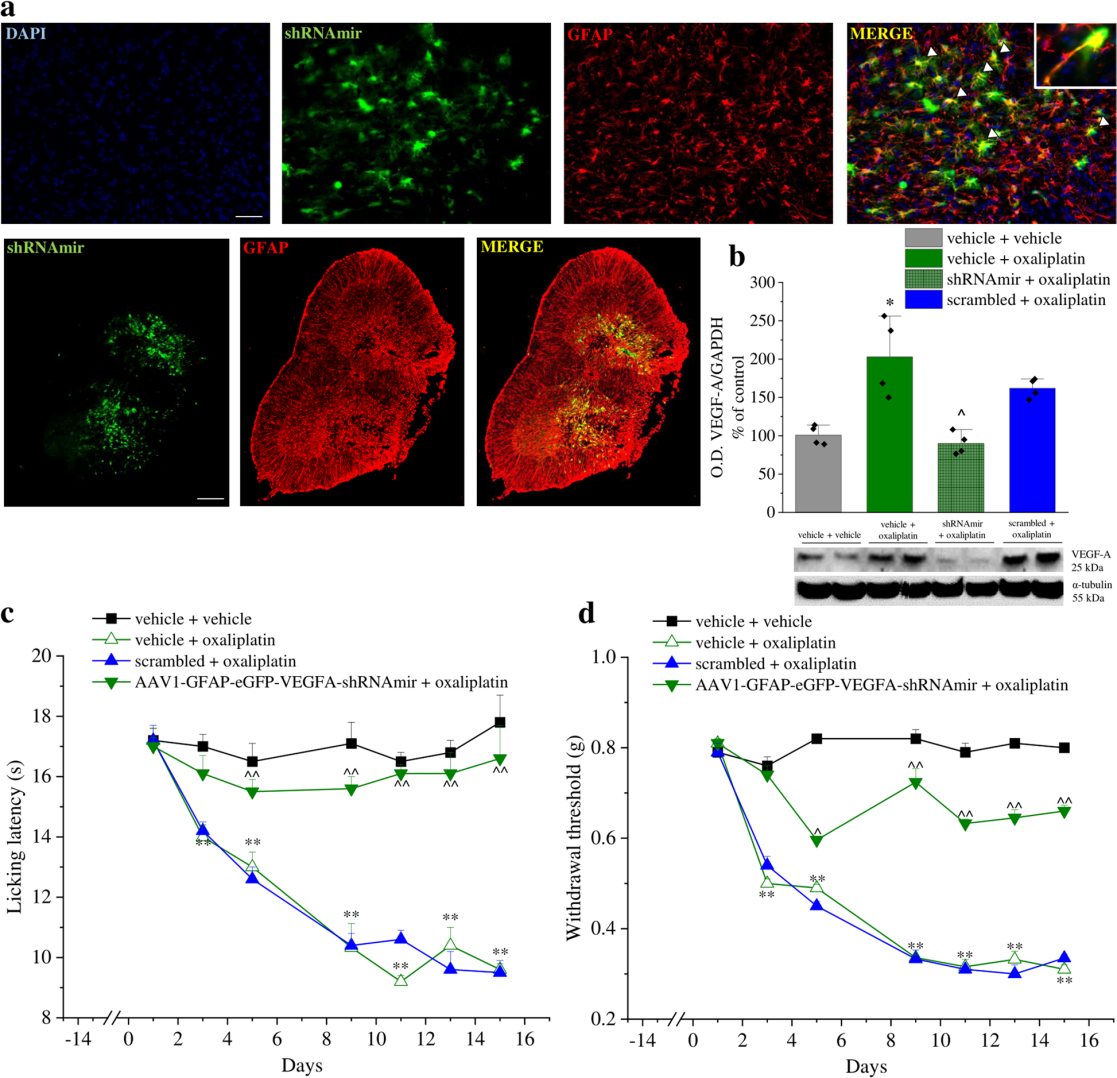
VEGF-A silencing in astrocytes prevents neuropathic pain. VEGF-A in spinal astrocytes was silenced by injecting in the spinal cord a VEGF-A specific AAV-derived shRNAmir targeting glial cells (AAV1-GFAP-eGFP-VEGF-A-shRNAmir). A shRNAmir vector containing a scrambled sequence was injected and used as control. (**a**) Representative images of eGFP and GFAP fluorescence in a whole section at lumbar level, scale bar: 100 μm. Higher magnifications were reported to visualize the colocalization, scale bar: 50 μm (*n* = 4). (**b**) Representative western blot images and densitometric analysis showing the expression of VEGF-A in the lumbar section of the spinal cord after vector administration (n = 4, blot of samples obtained from 2 animals of each group are shown). Pain threshold was evaluated by (**c**) Cold plate and (**d**) Paw pressure tests (n = 5). Each value represents the mean ± SEM. *P < 0.05 and **P < 0.01 vs vehicle; ^P < 0.05 and ^^P < 0.01 vs scrambled + oxaliplatin group. The analysis of variance was performed by one-way ANOVA. A Bonferroni’s significant difference procedure was used as post-hoc comparison

To analyse the influence of astrocytic VEGF-A modulation on pain signalling, we selectively silenced VEGF-A in spinal astrocytes by injecting a VEGF-A specific AAV-derived shRNAmir targeting glial cells. The vector was bilaterally injected at the lumbar and thoracic levels of the spinal cord 2 weeks before the first oxaliplatin treatment. As shown in Fig. 7a, 4 weeks after injection, the vector fluorescence colocalized with GFAP-positive cells inducing a significant decrease of VEGF-A expression (Fig. 7b). The pain threshold measurements, by employing thermal (Cold plate test) and mechanical (von Frey test) non-noxious stimuli over time, showed a significant prevention of hypersensitivity development during the 2 weeks of oxaliplatin treatment in the group that received the VEGF-A specific shRNAmir in comparison to scrambled- and vehicle-treated mice (Fig. 7c and d). To verify the lack of neurological and motor alterations which could interfere with pain behaviour recordings, the motor functionality and exploratory activity of VEGF-A shRNAmir and scrambled-treated mice were evaluated by the Hole board test. No alterations were highlighted with the exception of a higher exploratory activity on day 3 of oxaliplatin protocol (Table S2).

### The anti VEGFR-1 mAb D16F7 relieves pain in different models of CIN

**Fig. 8:**
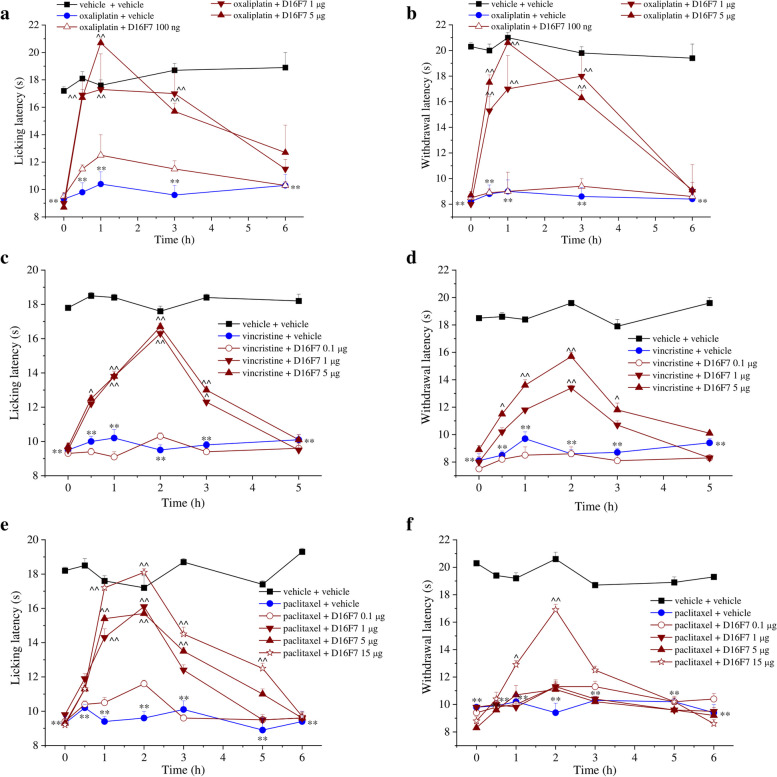
D16F7 mAb reduces pain in different models of chemotherapy-induced neuropathy. Effect of D16F7 mAb evaluated by (**a**) Cold plate and (**b**) Paw pressure tests in a mouse model of oxaliplatin-induced neuropathy after i.t. injection (a, b, *n* = 6). (**c**, **d**) Effect of D16F7 mAb after i.t. administration in vincristine-treated mice stimulated with thermal (**c**) or mechanical (**d**) stimuli (n = 6). (**e**, **f**) Effect of D16F7 after i.t. administration in paclitaxel-treated mice stimulated with thermal (**e**) or mechanical (**f**) stimuli (n = 6). Each value represents the mean ± SEM. **P < 0.01 vs vehicle + vehicle-treated animals; ^P < 0.05 and ^^P < 0.01 vs chemotherapeutic drugs + vehicle-treated animals. The analysis of variance was performed by one-way ANOVA. A Bonferroni’s significant difference procedure was used as post-hoc comparison

To investigate the therapeutic role of a pharmacological treatment targeting VEGFR-1 in the management of neuropathic pain induced by anticancer drugs, animals with oxaliplatin-induced neuropathy were treated with the anti-VEGFR-1 mAb D16F7. In this model, i.t. infusion of D16F7 (100 ng, 1 μg and 5 μg) induced a significant, dose-dependent, increase of the pain threshold both after thermal and mechanical non-noxious and noxious stimulation. Hypersensitivity was fully counteracted (up to control values) from 30 min to 3 h after treatment (Fig. 8a and b). On the contrary, the anti VEGFR-2 antibody DC101 (100 pg i.t.) was ineffective (Fig. S7). Interestingly, D16F7 mAb maintained its efficacy also when systemically injected by the i.p. route (1, 5, 15 and 25 mg kg^− 1^), starting from the dose of 5 mg kg^− 1^. The onset of the analgesic effect was observed at 60 min, and efficacy was maintained up to 120 min (Fig. S8). The pain-relieving properties of D16F7 mAb seem not to be limited to the oxaliplatin neurotoxicity since it was also effective in mice which become hypersensitive after treatment with the neurotoxic anticancer drugs vincristine and paclitaxel. In both models, D16F7 mAb (1 and 5 μg, i.t.) was active between 30 min and 3 h (Fig. 8c-f) in the Cold plate and Paw pressure tests with a particular efficacy when the pain response was evoked by thermal stimuli (Fig. 8c and e). In paclitaxel-treated mice, 15 μg D16F7 mAb dosed i.t. were effective up to 5 h (Fig. 8e).

## Discussion

Our data indicate that VEGF-A evokes pain through VEGFR-1 activation in physiological and pathological conditions. In particular, CIN-related pain is sustained by a spinal VEGF-A release from astrocytes and can be counteracted by local or systemic administration of the anti-VEGFR-1 D16F7, a mAb also endowed with anti-angiogenic and antitumor activity.

Using recombinant VEGF_165_b, we showed an increase of the electrophysiological activity of nociceptive neurons in the spinal cord with a consequent, significant decrease of the pain threshold. These data are in agreement with our previous results [4] and with the peripheral pro-nociceptive effect of VEGF-A demonstrated by Selvaraj and colleagues [6] after an intraplantar injection as well as with the VEGF-A increase in synovial fluid of subjects afflicted by osteoarticular pain [7]. Furthermore, in the peripheral nervous system, an anti-VEGF-A mAb treatment was found to alleviate neuropathic pain induced by the chronic constriction injury of the sciatic nerve [5], suggesting an active role of VEGF-A also in the peripheral sensitization mechanisms [63]. In our hands, both the selective VEGFR-1 agonist PlGF-2 and the selective VEGFR-2 agonist VEGF-E induced nociception after i.t. infusion. In addition, the selective anti-VEGFR-2 mAb DC101 induced hypersensitivity whereas the selective anti-VEGFR-1 mAb D16F7 did not. Based on these data, it could be hypothesized that VEGFR-1 selectively mediates pain, either directly stimulated by exogenously PlGF-2 or the endogenously produced VEGF-A displaced from VEGFR-2 by VEGF-E or the competitive DC101 mAb. In this context, it is worth noting that D16F7 mAb is a non-competitive inhibitor that hampers VEGFR-1 activation without affecting ligand binding [32, 33]. The involvement of VEGFR-1 in algesia induced by VEGF-A was confirmed by the ability of D16F7 mAb to block the nociceptive effects of all the agonists and of DC101 mAb. Furthermore, the knockdown of VEGFR-1 prevented VEGF_165_b, PlGF-2 and VEGF-E effects, strongly supporting the pivotal role of this receptor in the spinal pain pathways. These data are in agreement with those described by Selvaraj and colleagues [6] in the peripheral nervous system where VEGF-A induced nociceptive sensitization via VEGFR-1. Consistently with behavioural data, electrophysiological experiments revealed that VEGF_165_b spinal application, caused a marked increase in both spontaneous and evoked activity of NS neurons in naïve animals. In particular, the increased responsiveness to mechanical noxious stimuli of NS neurons induced by VEGF_165_b spinal microinjection suggests that low doses of this compound were able to induce a central sensitisation, similar to the neuropathic pain condition induced by nerve injury. In this context, the pre-application of D16F7 prevented the VEGF-A-induced neuronal hyperexcitability, confirming the contribution of this receptor in VEGF-A-mediated painful effects.

In the normal healthy CNS, VEGF-A regulates microvascular density, vessel permeability, and maintains endothelial cell fenestration in the choroid plexus, stimulates neural stem cell proliferation and promotes neurogenesis [25]. In pathological conditions (besides beneficial vascular effects), it safeguards stressed neurons, induces axon extension and branching, and promotes synaptic plasticity; furthermore, VEGF-A triggers proliferation, survival and migration of astrocytes and stimulates expression of trophic factors by astrocytes and microglia [23, 25]. Glial cells play a crucial role in the maladaptive plasticity of the nervous system in chronic pain and, particularly, in neuropathies [64]. Activated by neuronal damage or by signals from periphery, glia participates in pain development and chronicization, amplifying the excitatory synaptic microenvironment [65, 66]. Released soluble factors, like cytokines and growth factors, possess a direct nociceptive effect [67]. Among the latter, NGF, BDNF, GDNF, and other factors, seem unable to separate the neuroprotective effect from the algic one, probably following the evolutionary positive alarm role of physiological pain [68–70]. In this context, the nociceptive effect of VEGF-A is not surprising. Neuropathies induced by trauma to a peripheral nerve [11] or by chemotherapy [4] are characterized by enhanced spinal concentration of VEGF-A. The present results show, as expected [25], not only a relevant spinal VEGF-A concentration in the vessel structures, but also the existence of an *extra*-endothelial component. In comparison to microglia and neurons, astrocytes of healthy mice showed the highest amount of VEGF-A, which was clearly distinguishable from the vascular component. The repeated treatment with oxaliplatin up to the development of painful neuropathy significantly enhanced the presence of the growth factor in astroglia. The selective VEGF-A knockdown in dorsal horn astrocytes at the lumbar and thoracic levels of the spinal cord strongly reduced oxaliplatin-dependent neuropathic pain suggesting astrocytic VEGF-A as a relevant component of the pain signalling orchestrated by the glia. Furthermore, enhanced concentrations of VEGF-A can also lead to other pathological alterations related to neuropathies like an increased BBB permeability [71–73]. In fact, while low basal levels of VEGF-A are necessary for the maintenance of BBB integrity, high levels of the growth factor can alter BBB permeability and compromise CNS functions [26, 27]. The hypoxia inducible factor-1 driven by IL-1 promotes VEGF-A release from astrocytes that induces down-regulation or loss of the endothelial tight junction proteins claudin-5 and occludin, determining a loss of BBB function [26, 74] by mechanisms involving VEGFR-1 [8]. On the other hand, the increase in VEGF-A levels in neurotoxic conditions is generally related to hypoxia, as clearly demonstrated in diabetic and chemotherapy-induced neuropathies [4, 12, 75], suggesting the need of improving vascular functions [25]. The rescue role of VEGF-A is also based on its extra-vascular neuroprotective and neuroregenerative properties mainly due to the activation of the VEGFR-2. VEGF-A stimulates the migration and survival of Schwann cells [76] and protects neurons against chemotherapy-induced cytotoxicity via activation of VEGFR-2 and MEK1/2 and inhibition of caspase3 [77]. VEGF-A-signalling through VEGFR-2 leads to the protection of dorsal root ganglion sensory neurons in models of drug (paclitaxel) or hyperglycaemia-induced neuropathies, through induction of Heat Shock Protein 90 deacetylation and increase of Bcl-2 [9, 10]. The loss of endothelial VEGFR-2 signalling leads to tissue alteration in the dorsal horn and the development of hyperalgesia whereas neuronal overexpression of VEGFR-2 in mice reduced the sensitivity to paclitaxel-induced peripheral neuropathy [9]. This outcome seems to be related to neuroprotective effects and, accordingly, we showed an increase of VEGFR-2 spinal expression in oxaliplatin-treated mice that could be considered an adaptive response to the damage. On the contrary, the acute stimulation of VEGFR-2 does not directly interfere with pain.

Our data show that VEGF-A induces pain by selectively activating the VEGFR-1, which is expressed on spinal sensory neurons. A dichotomy between the pro-algesic VEGFR-1-signaling and the protective VEGFR-2-signaling is suggested, offering the possibility to relieve pain through a target that conserves the neuroprotective effects of the endogenous VEGF-A. In this view, the selective anti-VEGFR-1 mAb D16F7 induced a potent pain-relieving effect against nociception triggered by VEGF-A or PlGF-2, as well as against neuropathic pain evoked by the neurotoxic adverse reactions of different anticancer drugs like oxaliplatin, paclitaxel and vincristine. In addition, the pain-relieving effect of D16F7 was demonstrated after local (i.t.) and systemic (i.p.) administration. D16F7 mAb is able to inhibit VEGFR-1 homodimerization, auto-phosphorylation and downstream signal transduction [33–35] and down-modulates membrane receptor signalling without affecting VEGF-A or PlGF binding [32, 33]. Indeed, D16F7 mAb interacts with a receptor site corresponding to amino acids 149–161 of human VEGFR-1, which is different from that involved in VEGF-A or PlGF binding [33, 78, 79].

CIN is one of the most common adverse events of several first-line chemotherapeutic agents, affecting several million patients worldwide each year and reducing the benefits of effective anticancer therapies in the long-term outcome. It is not possible to predict which patients will develop symptoms and when  the latter will occurr during the chemotherapy course. Moreover, pain and sensory abnormalities may persist for months, or even years after the cessation of chemotherapy [80, 81]. The management of chemotherapy-induced neuropathy is a significant challenge and there are no drugs approved to prevent or alleviate CIN [3]. In this scenario, VEGF-A is candidate to be a possible plasmatic biomarker [4] strictly related to pain and the selective blockade of VEGFR-1 is proposed as a theoretically ideal strategy to relieve neuropathic hypersensitivity. D16F7 is effective and potent against pain induced by several neurotoxic anticancer drugs; it is active by a systemic route of administration that allows to reach therapeutic concentrations of the mAb also at the CNS level, as demonstrated by its antitumor efficacy in orthotopic preclinical models of glioblastoma [35]. Thus, D16F7 possesses a double effective profile that could be evaluated in the future in tumour-bearing mouse models of CIN; this would allow to overcome the current limitations of this research study that was conducted in animals without cancer. Finally, D16F7 offers the safety qualities requested for the pharmacological treatment of cancer patients in the presence of a possible co-treatment with chemotherapy. The VEGFR-1 target is mostly involved in pathological processes rather than in physiological conditions [32] and in preclinical in vivo studies repeated dosing schedules of the anti-VEGFR-1 D16F7 did not cause significant adverse effects, both as single agent or in combination with immune checkpoint inhibitors [28, 33, 35, 36]. Interestingly, due to the non-competitive antagonism at the membrane receptor level, D16F7 does not interfere with ligand binding and with the decoy function of soluble VEGFR-1, represented by shorter receptor forms lacking the transmembrane and intracellular regions. Soluble VEGFR-1 is released in the extra-cellular matrix and is capable of sequestering PlGF or VEGF-A, preventing their interaction with the membrane receptor [32]. Thus, in the presence of D16F7, the anti-angiogenic, anti-oedema and anti-inflammatory properties of soluble VEGFR-1 would be preserved, contributing to the further control of neuropathic pain. The unveiling of the downstream receptor signalling involved in the complex pain relieving mechanisms [82, 83] deserves further investigation.

## Conclusions

In conclusion, VEGF-A is a pro-nociceptive molecule that activates neuronal firing and induces pain by VEGFR-1 stimulation. Interestingly, VEGF-A increases during CIN, and its release from spinal astrocytes plays a decisive role in neuropathic pain development. Moreover, the anti-VEGFR-1 mAb D16F7 is suggested as a promising candidate for the treatment of CIN, adding this property to its previously described antitumor efficacy. The results of this proof of concept study encourage further investigation on the most effective therapeutic schedule for long-term pain control.

### Supplementary Information

**Additional file 1: Table S1**. List of antibodies used for immunohistochemistry and western blot assays.

**Additional file 2: Figure S1**. Nociceptive effect of VEGF_165_a. The pain threshold was measured by the Cold plate test over time after intrathecal injection of VEGF_165_a (1-30 ng; *n*=5). Each value represents the mean ± SEM. **P<0.01 vs vehicle-treated animals. The analysis of variance was performed by one-way ANOVA. A Bonferroni’s significant difference procedure was used as post-hoc comparison.

**Additional file 3: Figure S2**. Hypersensitivity-induced by VEGF-A family members is not due to the interaction with VEGFR-2. The response to a thermal stimulus (Cold plate test) was recorded after intrathecal infusion (30 ng) of (a) VEGF_165_b (n=5), (b) PlGF-2 (n=5), (c) VEGF-E (n=5), following pre-treatment (15 min before) with vehicle or anti-VEGFR-2 mAb DC101 (100 pg). Each value represents the mean ± SEM. **P<0.01 vs vehicle + vehicle-treated animals. The analysis of variance was performed by one-way ANOVA. A Bonferroni’s significant difference procedure was used as post-hoc comparison.

**Additional file 4: Figure S3**. DC101 increases spontaneous and noxious-evoked activity of NS neurons. Representative ratematers showing spontaneous and noxious-evoked activity of NS neurons after spinal application of DC101 antibodies at 100 pg (a) and 30 pg (b). Black arrows indicate the noxious stimulation on the mouse hind-paw. Mean ± SEM population data of spinal cord application of DC101 (30 pg and 100 pg) on % variation of firing rate (c), % variation of frequency of excitation (d) and % variation of duration of evoked activity (e) of NS neurons in CD1 mice. Black arrows indicate vehicle, DC101 spinal application. Each point represents the mean of 5 different mice per group (one neuron recorded per each mouse). *P<0.05, **P<0.01 and ***P<0.001 indicate statistically difference vs pre-drug. One-way ANOVA followed by Dunnet’s multiple comparison post-hoc test was performed for statistical significance within groups.

**Additional file 5: Figure S4**. Analyses of GFAP and VEGF-A co-localization in confocal z-stacks. a) Cytofluorogram relative to images in Fig. 5. b) Li’s Intensity Correlation Analysis relative to images in Fig. 5. c) Van Steensel’s Cross-Correlation Function (CCF), relative to all datasets (*n*=8, mean ± SEM). Pearson’s correlation coefficient (PCC) is given by the CCF value corresponding to x =0. CCF at FWHM = 1.00 ± 0.04 μm (mean ± SEM, n=8). d, e). Analyses of AQP4 and VEGF-A co-localization in confocal z-stacks. D) Cytofluorogram relative to images in Fig. 5 e) Li’s Intensity Correlation Analysis relative to images in Fig. 5. CF Van Steensel’s Cross-Correlation Function (CCF), relative to all datasets (n=8, mean ± SEM). Pearson’s correlation coefficient (PCC) is given by the CCF value corresponding to x =0. CCF at FWHM =1.28±0.04 μm (mean ± SEM, n=8).

**Additional file 6: Figure S5**. VEGF-A is increased in the spinal cord of mice with oxaliplatin-induced neuropathy. Representative western blot images and densitometric analysis of VEGF-A expression in the lumbar section of the spinal cord of oxaliplatin-treated mice in comparison to control (*n*=4). Each value represents the mean ± SEM. *P<0.05 vs vehicle group. The analysis of variance was performed by one-way ANOVA. A Bonferroni’s significant difference procedure was used as post-hoc comparison.

**Additional file 7: Figure S6**. VEGFR-2 is increased in the spinal cord of mice with oxaliplatin-induced neuropathy. Representative western blot images and densitometric analysis of VEGF-R1 and VEGFR-2 expression in the lumbar section of the spinal cord of oxaliplatin-treated mice in comparison to control (n=4). Each value represents the mean ± SEM. *P<0.05 vs vehicle group. The analysis of variance was performed by one-way ANOVA. A Bonferroni’s significant difference procedure was used as post-hoc comparison.

**Additional file 8: Table S2.** Hole Board test. (DOCX 17 kb). The Hole board test was performed 3, 5 and 9 days after the beginning of oxaliplatin treatment (*n*=5). Each value represents the mean ± SEM. **P<0.01 vs vehicle + vehicle treated animals. The analysis of variance was performed by one-way ANOVA. A Bonferroni’s significant procedure was used as post-hoc comparison.

**Additional file 9: Figure S7.** DC101 mAb does not reduce pain in oxaliplatin-induced neuropathic pain model. Effect of DC101 mAb evaluated by Cold plate test in a mouse model of oxaliplatin-induced neuropathy after i.t. injection (*n*=6). Each value represents the mean ± SEM. **P<0.01 vs vehicle + vehicle-treated animals. The analysis of variance was performed by one-way ANOVA. A Bonferroni’s significant difference procedure was used as post-hoc comparison.

**Additional file 10: Figure S8**. D16F7 mAb reduces oxaliplatin-induced pain after systemic administration. Effect of D16F7 mAb evaluated by (a) Cold plate and (b) Paw pressure tests in a mouse model of oxaliplatin-induced neuropathy after i.p. injection (a, b, n = 6). Each value represents the mean ± SEM. **P < 0.01 vs vehicle + vehicle-treated animals; ^^P < 0.01 vs oxaliplatin + vehicle-treated animals. The analysis of variance was performed by one-way ANOVA. A Bonferroni’s significant difference procedure was used as post-hoc comparison.

## Data Availability

The data that support the findings of this study are available from the corresponding author upon reasonable request.

## Abbreviations

AAV: Adeno-associated virus
AQP-4: Aquaporin 4
BBB: Blood-brain-barrier
CIN: Chemotherapy-induced neuropathy
CNS: Central nervous system
GFAP: Glial fibrillary acidic protein
GFP: Green fluorescent protein
NS neurons: Nociceptive specific neurons
PlGF-2: Placental growth factor 2
VEGF-A: Vascular endothelial growth factor A
VEGFR: VEGF-A receptor
