# Correction: VEGF-A/VEGFR-1 signalling and chemotherapy-induced neuropathic pain: therapeutic potential of a novel anti-VEGFR-1 monoclonal antibody

**DOI:** 10.1186/s13046-024-03037-4

**Published:** 2024-04-23

**Authors:** Laura Micheli, Carmen Parisio, Elena Lucarini, Alessia Vona, Alessandra Toti, Alessandra Pacini, Tommaso Mello, Serena Boccella, Flavia Ricciardi, Sabatino Maione, Grazia Graziani, Pedro Miguel Lacal, Paola Failli, Carla Ghelardini, Lorenzo Di Cesare Mannelli

**Affiliations:** 1 Department of Neuroscience, Psychology, Drug Research and Child Health ‑ NEUROFARBA ‑ Pharmacology and Toxicology Section, University of Florence, Viale G. Pieraccini 6, 50139 Florence, Italy; 2 Department of Experimental and Clinical Medicine ‑ DMSC ‑ Anatomy and Histology Section, University of Florence, L.Go Brambilla 3, 50134 Florence, Italy; 3 Department of Biomedical, Experimental and Clinical Sciences, University of Florence, Viale G. Pieraccini 6, 50139 Florence, Italy; 4 Department of Experimental Medicine, Section of Pharmacology, University of Campania “L. Vanvitelli”, Via Santa Maria Di Costantinopoli 16, 80138 Naples, Italy; 5 I.R.C.S.S., Neuromed, 86077 Pozzilli, Italy; 6 Department of Systems Medicine, University of Rome Tor Vergata, Via Montpellier 1, 00133 Rome, Italy; 7 IDI-IRCCS, Via Monti di Creta 104, 00167 Rome, Italy

**Correction: J Exp Clin Cancer Res 40, 320 (2021)**

**https://doi.org/10.1186/s13046-021-02127-x**

Following publication of the original article [1], the authors identified an error in Fig. 6. The image representative of cell nuclei (DAPI staining) of the group “control” (panel “b”, first on the left) was unintentionally duplicated in panel “c” relative to the group “oxaliplatin” during the image assembly process by the authors.

The correction does not alter the scientific outcome since the result about the colocalization analysis of VEGF-A and GFAP in control and oxaliplatin-treated mice did not require the use of DAPI channel. The original article [1] has been corrected.

Incorrect Fig. 6

**Fig. 6:**
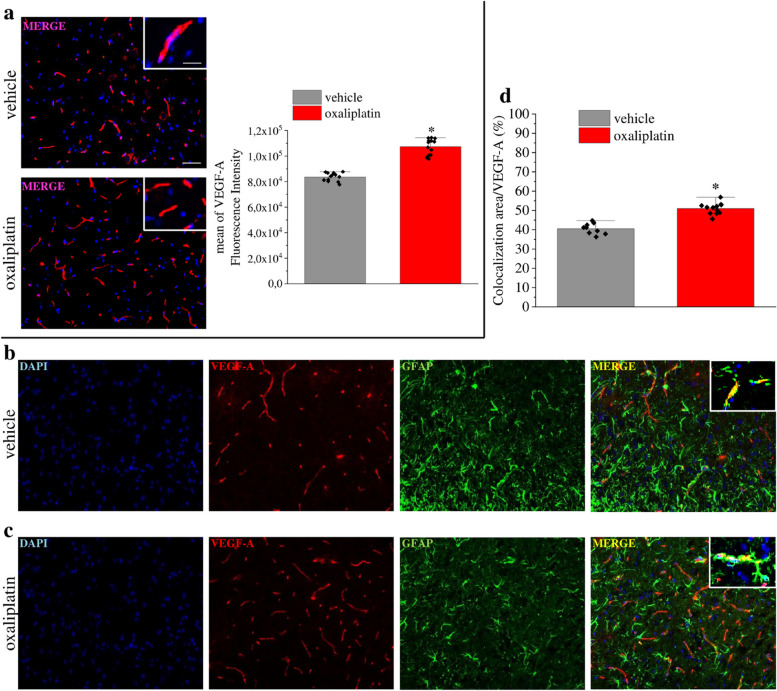
VEGF-A is increased in spinal astrocytes of mice with oxaliplatin-induced neuropathy. (**a**) Representative images and quantitative analysis of mean VEGF-A fluorescence intensity in the dorsal horn of oxaliplatin-treated mice in comparison to control animals (vehicle, *n* = 13). (**b-d**) Colocalization analysis of VEGF-A and GFAP in control (**b**) and oxaliplatin-treated mice (**c**). Quantitative analysis of colocalization area (**d**) (vehicle, n = 13; oxaliplatin, *n* = 12). Scale bar: 100 μm; insert: 50 μm. Each value represents the mean ± SEM. *P < 0.05 vs vehicle group. The analysis of variance was performed by one-way ANOVA. A Bonferroni’s significant difference procedure was used as post-hoc comparison

Correct Fig. 6

**Fig. 6:**
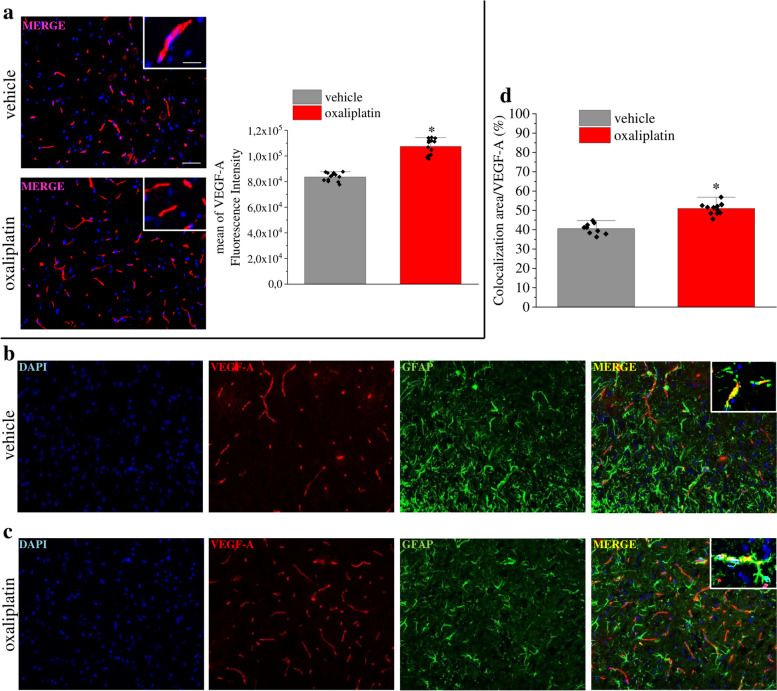
VEGF-A is increased in spinal astrocytes of mice with oxaliplatin-induced neuropathy. (**a**) Representative images and quantitative analysis of mean VEGF-A fluorescence intensity in the dorsal horn of oxaliplatin-treated mice in comparison to control animals (vehicle, *n* = 13). (**b-d**) Colocalization analysis of VEGF-A and GFAP in control (**b**) and oxaliplatin-treated mice (**c**). Quantitative analysis of colocalization area (**d**) (vehicle, n = 13; oxaliplatin, *n* = 12). Scale bar: 100 μm; insert: 50 μm. Each value represents the mean ± SEM. *P < 0.05 vs vehicle group. The analysis of variance was performed by one-way ANOVA. A Bonferroni’s significant difference procedure was used as post-hoc comparison
