# Supplementary material for: VEGF-A/VEGFR-1 signalling and chemotherapy-induced neuropathic pain: therapeutic potential of a novel anti-VEGFR-1 monoclonal antibody
Source: J Exp Clin Cancer Res. 2021 Oct 14;40:320. doi: 10.1186/s13046-021-02127-x (PMC8515680; doi:10.1186/s13046-021-02127-x)
Supplement: Supplementary file 1 — Additional file 1: Table S1. List of antibodies used for immunohistochemistry and western blot assays. [file 13046_2021_2127_MOESM1_ESM.docx]

**Supplementary Table S1. List of antibodies used for immunohistochemistry and western blot assays**

| **Target** | **Antigen** | **Supplier** | **Catalog#** | **Antibody** | **Host** | **Usage** | **Conc.** | **Analysis** |
| --- | --- | --- | --- | --- | --- | --- | --- | --- |
| Astrocytes | GFAP | Merck Millipore | MAB3402X | Monoclonal  conj. 488 | Ms | Primary | 1:500 | IF |
| Astrocytes | GFAP | Dako | ZO334 | Polyclonal | Rb | Primary | 1:500 | IF |
| Neurons | NeuN | Merck Millipore | MAB377X | Monoclonal  conj. 488 | Ms | Primary | 1:500 | IF |
| Microglia | Iba-1 | Wako | 016-20001 | Polyclonal | Rb | Primary | 1:200 | IF |
| Vascular Endothelial Growth Factor Receptor-1 | VEGFR1 | Bioss | bs-0170R | Polyclonal | Rb | Primary | 1:100 | IF |
| Vascular Endothelial Growth Factor-A | VEGF-A | Santa Cruz Biotechnology | sc-7269 | Monoclonal | Ms | Primary | 1:100 | IF |
| Vascular Endothelial Growth Factor | VEGF | BD Pharmigen | 555036 | Monoclonal | Ms | Primary | 1:1000 | WB |
| Vascular Endothelial Growth Factor Receptor-1 | VEGFR-1 | Abcam | 32152 | Monoclonal | Rb | Primary | 1:1000 | WB |
| Vascular Endothelial Growth Factor Receptor-2 | VEGFR2/DC101 | Bio X Cell | BE0060 | Monoclonal | Ms | Primary | 1:5000 | WB |
| Aquaporin 4 | AQP-4 | Santa Cruz Biotechnology | sc-32739 | Monoclonal | Ms | Primary | 1:100 | IF |
| RECA-1 | RECA-1 | Santa Cruz Biotechnology | Sc-52665 | Monoclonal | Ms | Primary | 1:100 | IF |
| Glyceraldehyde 3-phosphate dehydrogenase | GAPDH | Santa Cruz Biotechnology | sc-32233 | Monoclonal | Ms | Primary | 1:2500 | WB |
| Rabbit FC | Rabbit FC | Life technologies | A-11011 | Polyclonal | Rb | Secondary Alexa Fluor 568 | 1:500 | IF |
| Mouse FC | Mouse FC | Life technologies | A-11004 | Polyclonal | Ms | Secondary Alexa Fluor 568 | 1:500 | IF |
| Mouse FC | Mouse FC | Life technologies | A-11001 | Polyclonal | Ms | Secondary Alexa Fluor 488 | 1:500 | IF |
| Rabbit FC | Rabbit FC | Life technologies | A-21443 | Polyclonal | Rb | Secondary Alexa Fluor 647 | 1:200 | IF |
| 4’, 6-diamidin-2-fenilindolo | DAPI | Thermo scientific | 62248 | N.A. | N.A. | N.A. | 1:2000 | IF |
| Rabbit-HRP | r-IgG-h | Bethyl | A120-201P | Polyclonal | Rb | Secondary  conj. HRP | 1:5000 | WB |
| Mouse-HRP | m-IgGk BP-HRP | Santa Cruz Biotechnology | Sc-516102 | N.A. | Ms | Secondary  conj. HRP | 1:5000 | WB |
| α-tubulin | α-4a | Sigma-Aldrich | T6074 | Monoclonal | Ms | Secondary | 1:5000 | WB |
