# Supplementary figures and images for: VEGF-A/VEGFR-1 signalling and chemotherapy-induced neuropathic pain: therapeutic potential of a novel anti-VEGFR-1 monoclonal antibody
Source: J Exp Clin Cancer Res. 2021 Oct 14;40:320. doi: 10.1186/s13046-021-02127-x (PMC8515680; doi:10.1186/s13046-021-02127-x)

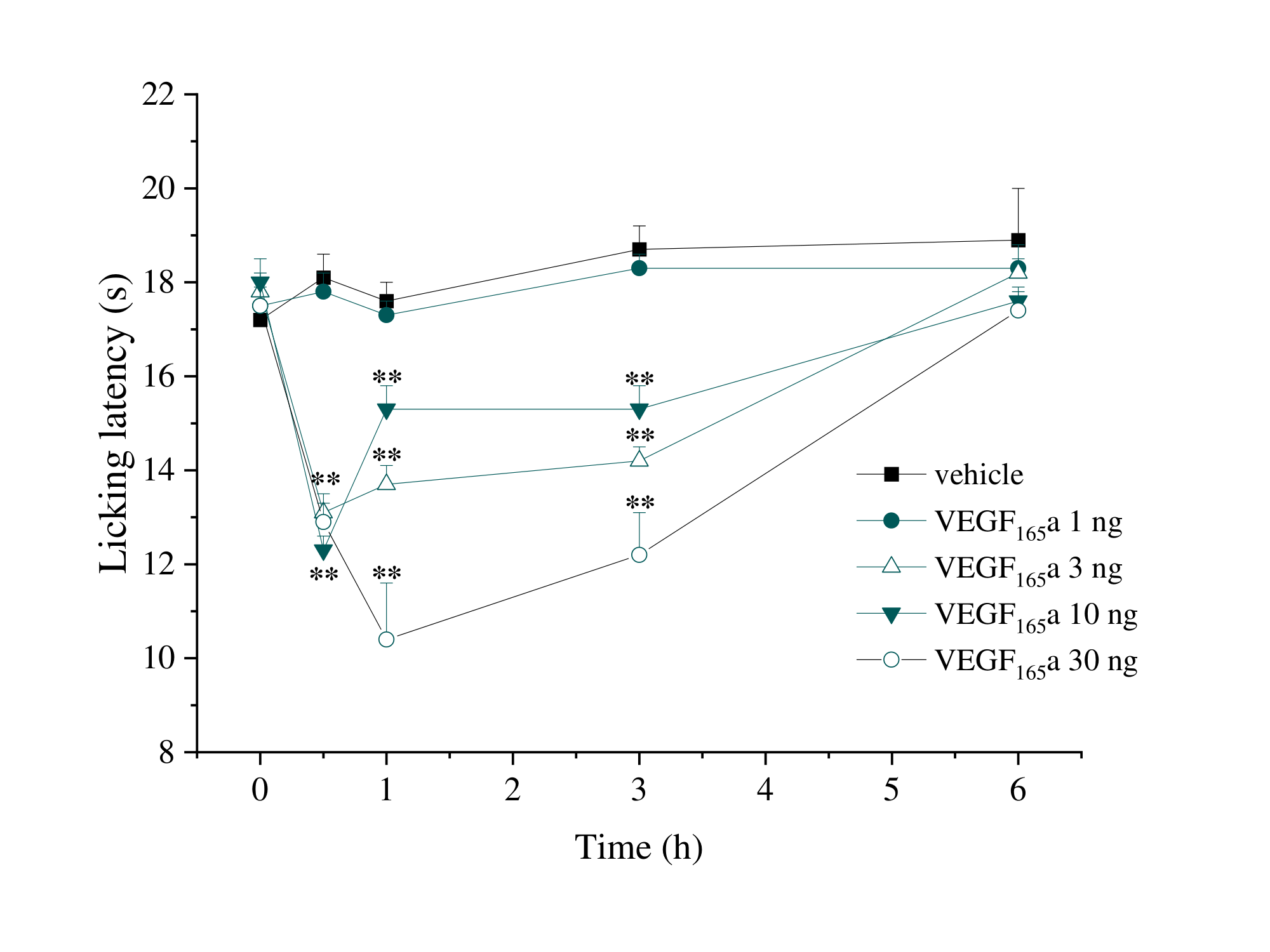

Supplement: Supplementary file 2 — Additional file 2: Figure S1. Nociceptive effect of VEGF165a. The pain threshold was measured by the Cold plate test over time after intrathecal injection of VEGF165a (1-30 ng; n=5). Each value represents the mean ± SEM. **P<0.01 vs vehicle-treated animals. The analysis of variance was performed by one-way ANOVA. A Bonferroni’s significant difference procedure was used as post-hoc comparison. [file 13046_2021_2127_MOESM2_ESM.tiff]

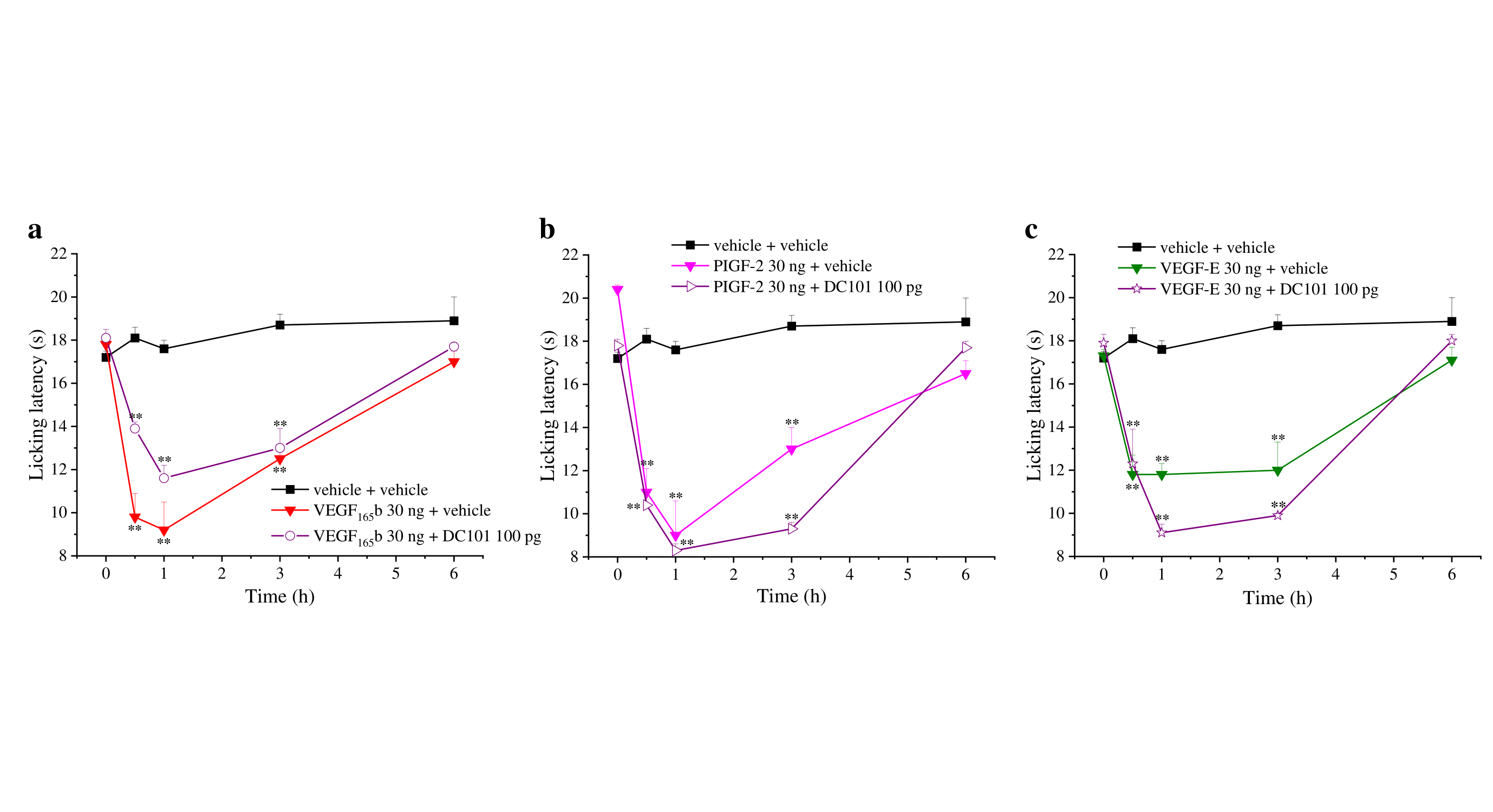

Supplement: Supplementary file 3 — Additional file 3: Figure S2. Hypersensitivity-induced by VEGF-A family members is not due to the interaction with VEGFR-2. The response to a thermal stimulus (Cold plate test) was recorded after intrathecal infusion (30 ng) of (a) VEGF165b (n=5), (b) PlGF-2 (n=5), (c) VEGF-E (n=5), following pre-treatment (15 min before) with vehicle or anti-VEGFR-2 mAb DC101 (100 pg). Each value represents the mean ± SEM. **P<0.01 vs vehicle + vehicle-treated animals. The analysis of variance was performed by one-way ANOVA. A Bonferroni’s significant difference procedure was used as post-hoc comparison. [file 13046_2021_2127_MOESM3_ESM.tiff]

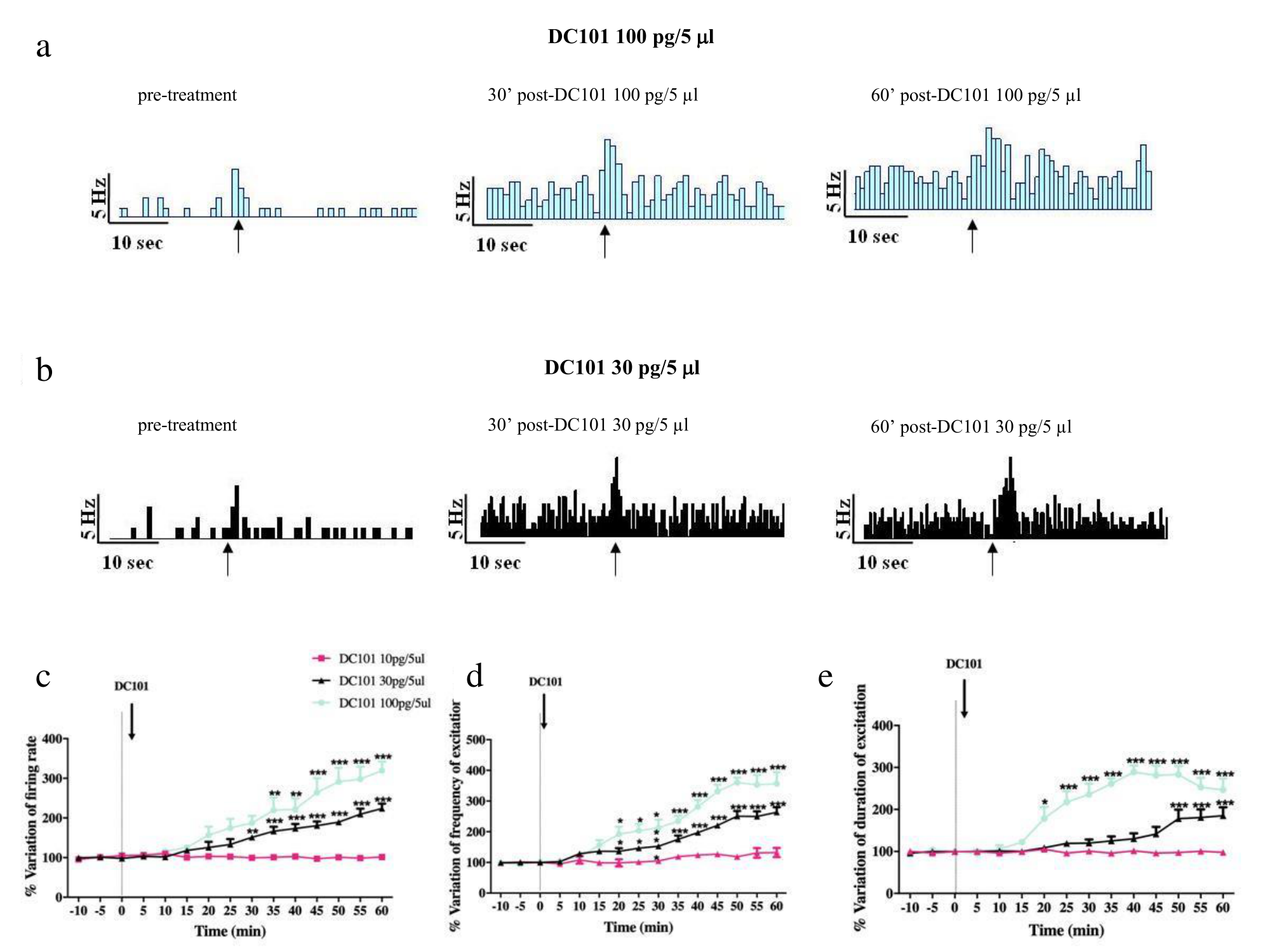

Supplement: Supplementary file 4 — Additional file 4: Figure S3. DC101 increases spontaneous and noxious-evoked activity of NS neurons. Representative ratematers showing spontaneous and noxious-evoked activity of NS neurons after spinal application of DC101 antibodies at 100 pg (a) and 30 pg (b). Black arrows indicate the noxious stimulation on the mouse hind-paw. Mean ± SEM population data of spinal cord application of DC101 (30 pg and 100 pg) on % variation of firing rate (c), % variation of frequency of excitation (d) and % variation of duration of evoked activity (e) of NS neurons in CD1 mice. Black arrows indicate vehicle, DC101 spinal application. Each point represents the mean of 5 different mice per group (one neuron recorded per each mouse). *P<0.05, **P<0.01 and ***P<0.001 indicate statistically difference vs pre-drug. One-way ANOVA followed by Dunnet’s multiple comparison post-hoc test was performed for statistical significance within groups. [file 13046_2021_2127_MOESM4_ESM.tiff]

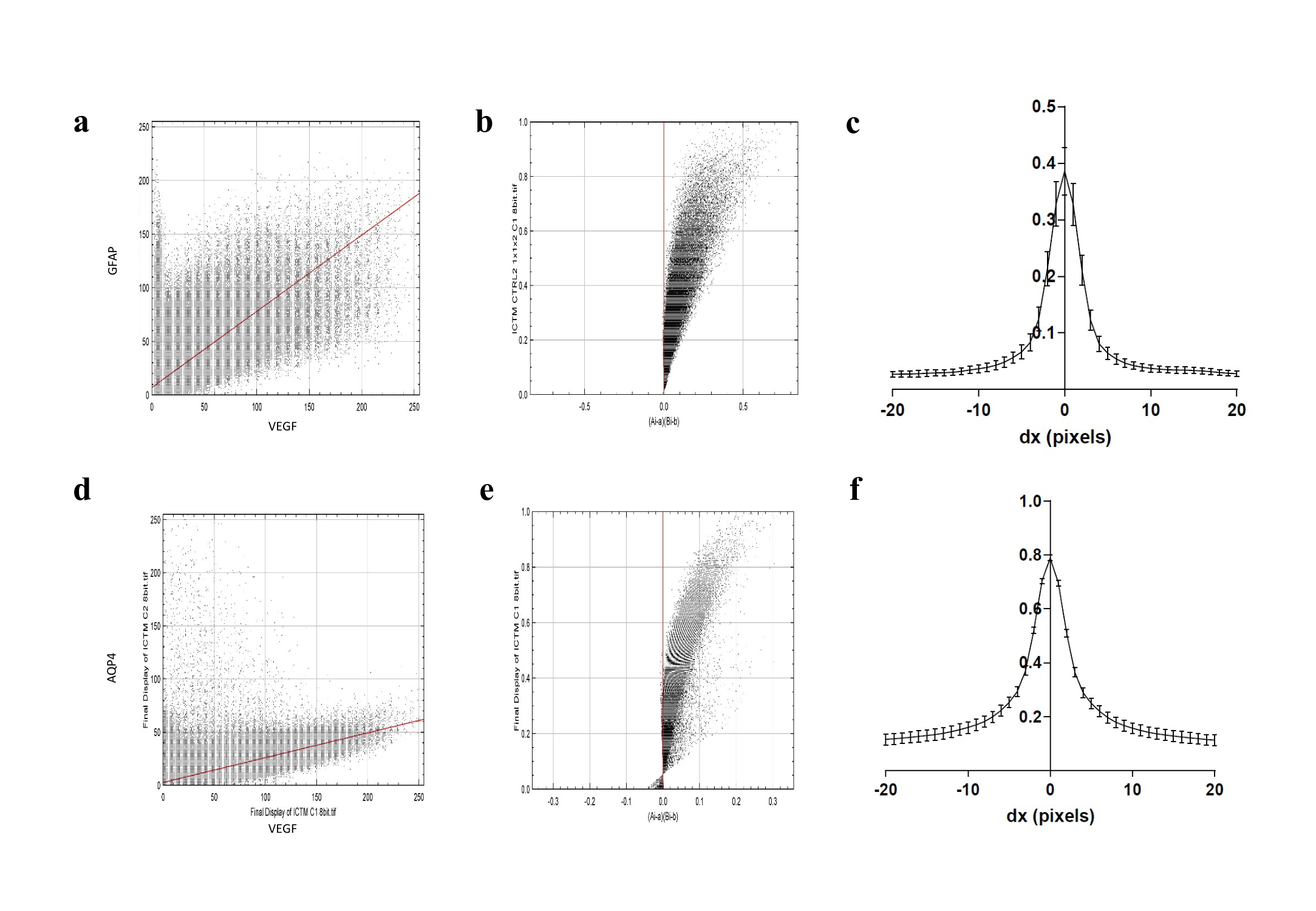

Supplement: Supplementary file 5 — Additional file 5: Figure S4. Analyses of GFAP and VEGF-A co-localization in confocal z-stacks. a) Cytofluorogram relative to images in Fig. 5. b) Li’s Intensity Correlation Analysis relative to images in Fig. 5. c) Van Steensel’s Cross-Correlation Function (CCF), relative to all datasets (n=8, mean ± SEM). Pearson’s correlation coefficient (PCC) is given by the CCF value corresponding to x =0. CCF at FWHM = 1.00 ± 0.04 μm (mean ± SEM, n=8). d, e). Analyses of AQP4 and VEGF-A co-localization in confocal z-stacks. D) Cytofluorogram relative to images in Fig. 5 e) Li’s Intensity Correlation Analysis relative to images in Fig. 5. CF Van Steensel’s Cross-Correlation Function (CCF), relative to all datasets (n=8, mean ± SEM). Pearson’s correlation coefficient (PCC) is given by the CCF value corresponding to x =0. CCF at FWHM =1.28±0.04 μm (mean ± SEM, n=8). [file 13046_2021_2127_MOESM5_ESM.tiff]

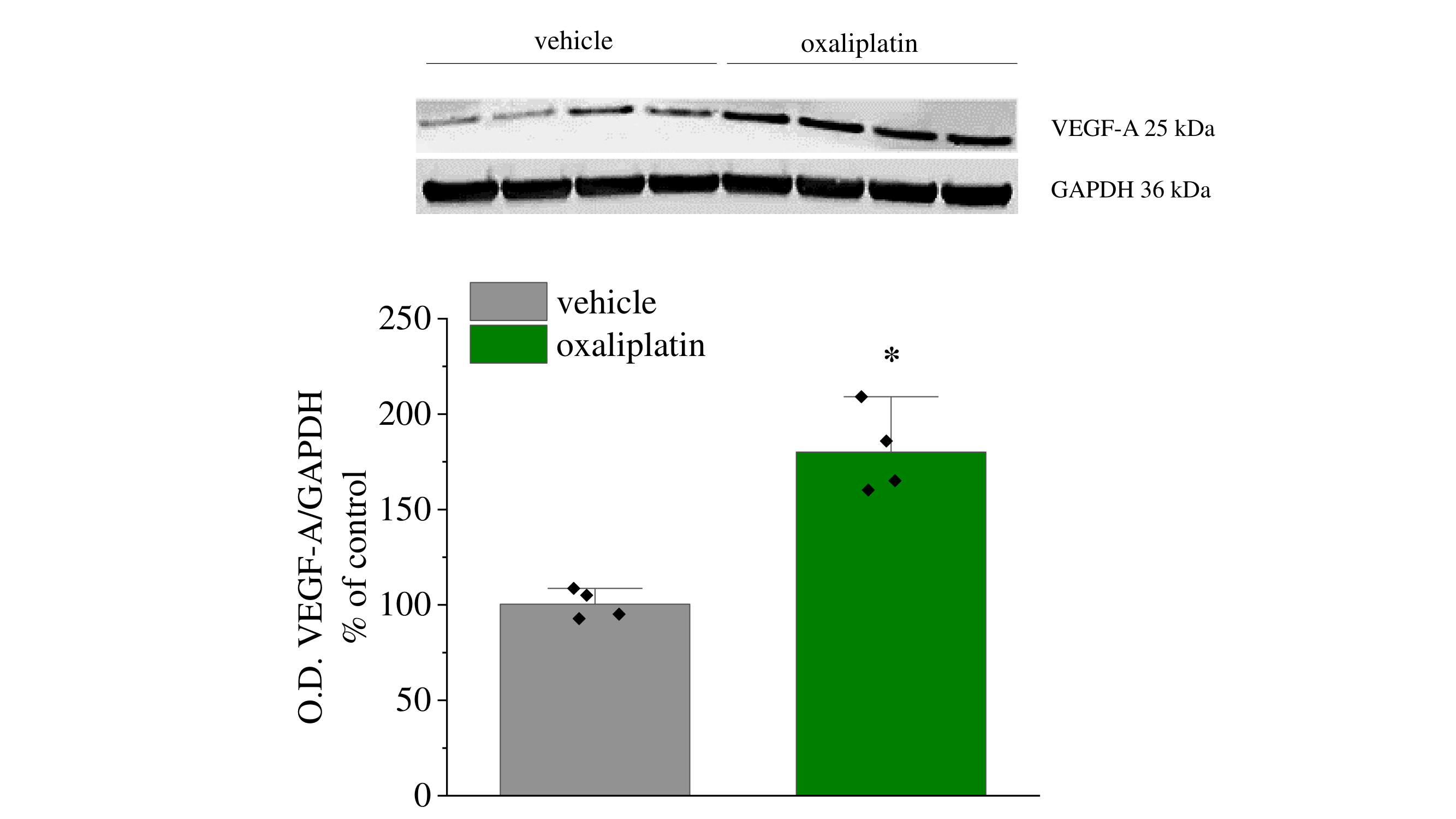

Supplement: Supplementary file 6 — Additional file 6: Figure S5. VEGF-A is increased in the spinal cord of mice with oxaliplatin-induced neuropathy. Representative western blot images and densitometric analysis of VEGF-A expression in the lumbar section of the spinal cord of oxaliplatin-treated mice in comparison to control (n=4). Each value represents the mean ± SEM. *P<0.05 vs vehicle group. The analysis of variance was performed by one-way ANOVA. A Bonferroni’s significant difference procedure was used as post-hoc comparison. [file 13046_2021_2127_MOESM6_ESM.tiff]

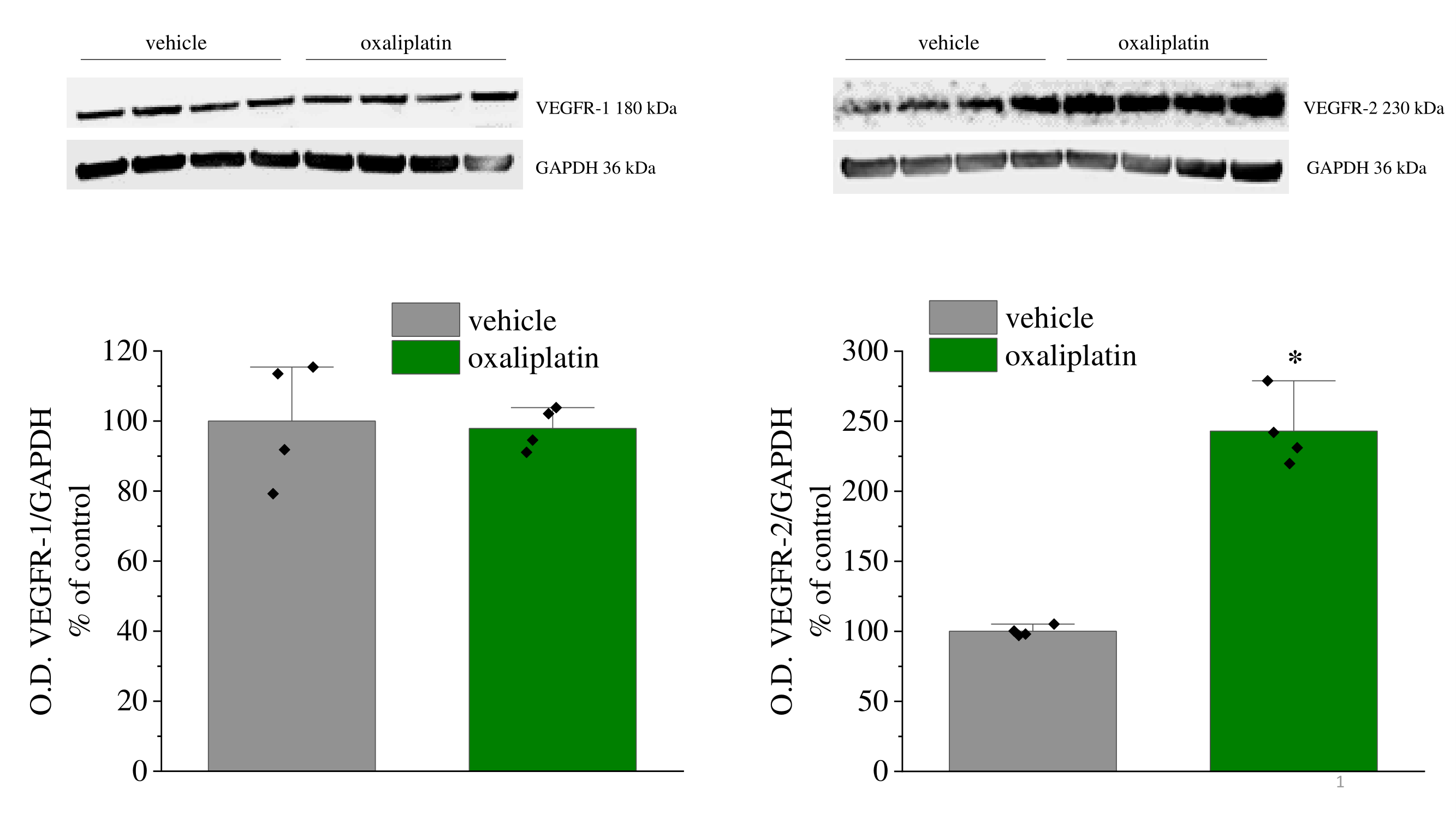

Supplement: Supplementary file 7 — Additional file 7: Figure S6. VEGFR-2 is increased in the spinal cord of mice with oxaliplatin-induced neuropathy. Representative western blot images and densitometric analysis of VEGF-R1 and VEGFR-2 expression in the lumbar section of the spinal cord of oxaliplatin-treated mice in comparison to control (n=4). Each value represents the mean ± SEM. *P<0.05 vs vehicle group. The analysis of variance was performed by one-way ANOVA. A Bonferroni’s significant difference procedure was used as post-hoc comparison. [file 13046_2021_2127_MOESM7_ESM.tiff]

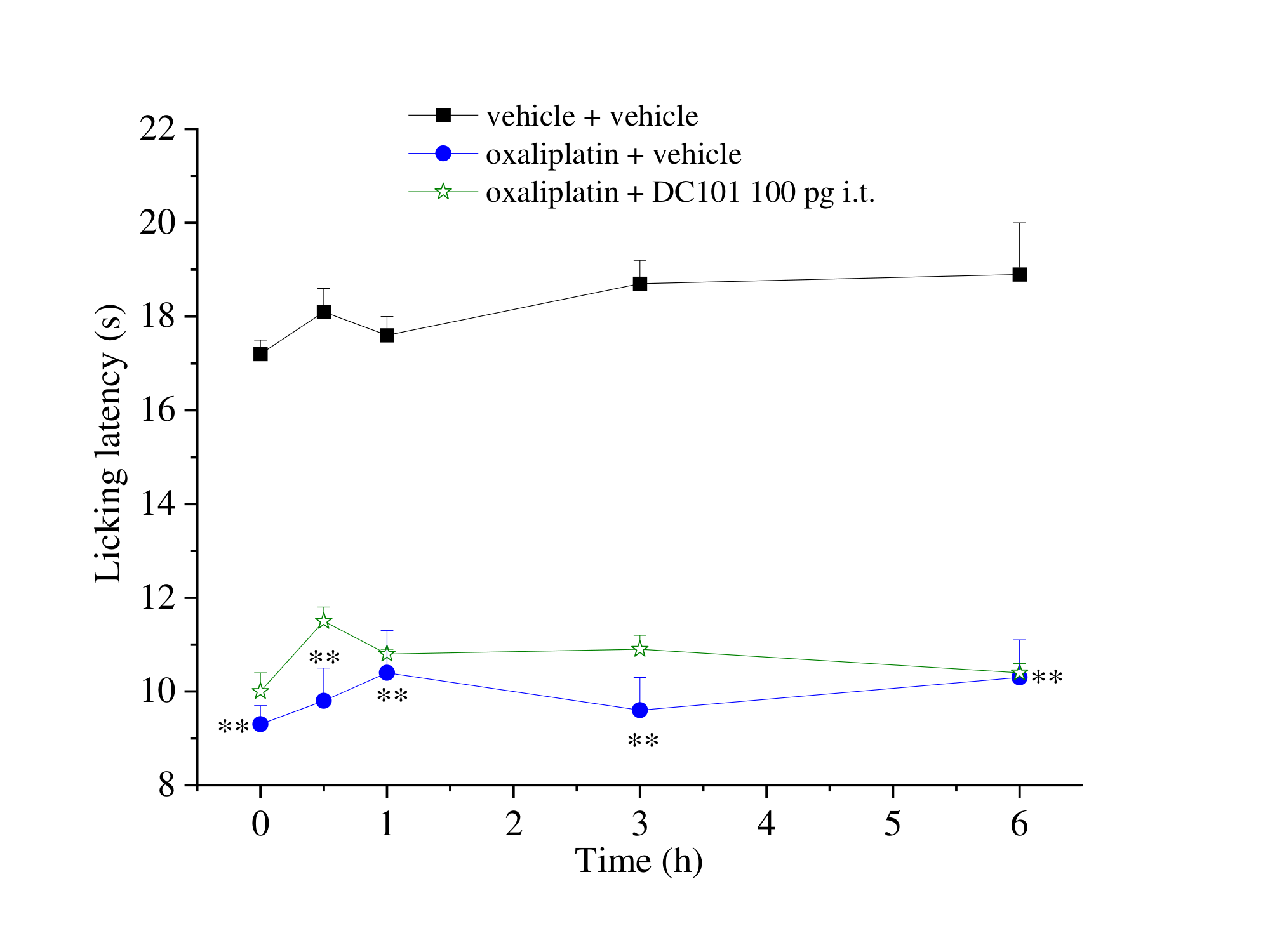

Supplement: Supplementary file 9 — Additional file 9: Figure S7. DC101 mAb does not reduce pain in oxaliplatin-induced neuropathic pain model. Effect of DC101 mAb evaluated by Cold plate test in a mouse model of oxaliplatin-induced neuropathy after i.t. injection (n=6). Each value represents the mean ± SEM. **P<0.01 vs vehicle + vehicle-treated animals. The analysis of variance was performed by one-way ANOVA. A Bonferroni’s significant difference procedure was used as post-hoc comparison. [file 13046_2021_2127_MOESM9_ESM.tiff]

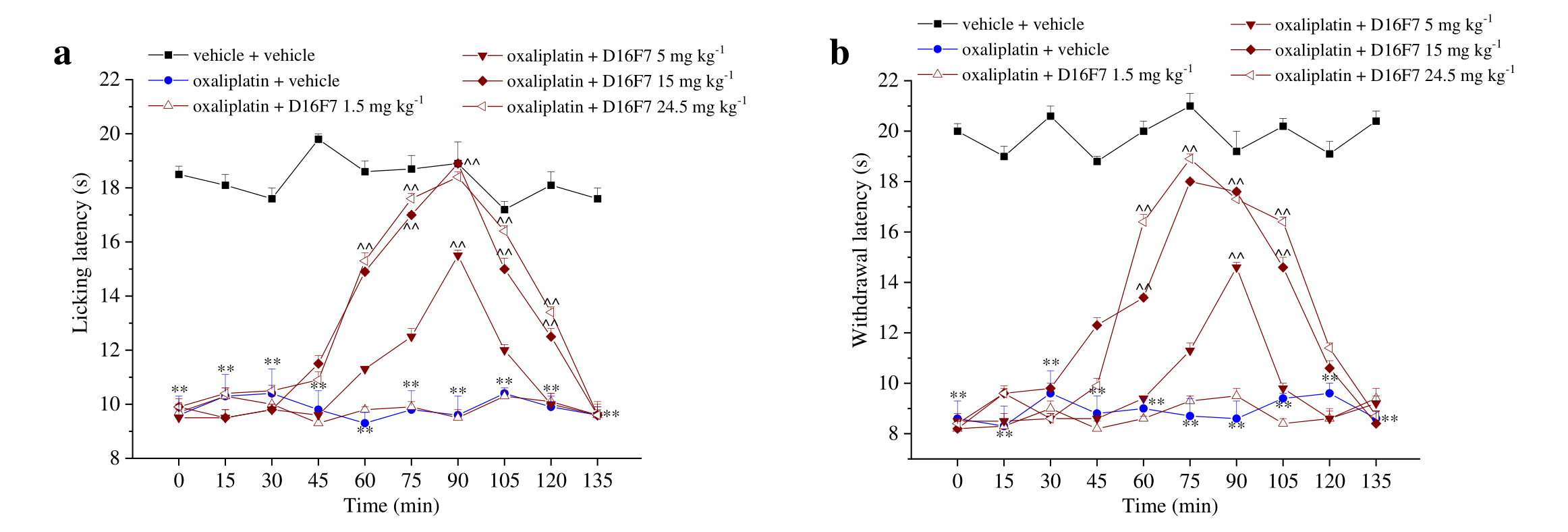

Supplement: Supplementary file 10 — Additional file 10: Figure S8. D16F7 mAb reduces oxaliplatin-induced pain after systemic administration. Effect of D16F7 mAb evaluated by (a) Cold plate and (b) Paw pressure tests in a mouse model of oxaliplatin-induced neuropathy after i.p. injection (a, b, n = 6). Each value represents the mean ± SEM. **P < 0.01 vs vehicle + vehicle-treated animals; ^^P < 0.01 vs oxaliplatin + vehicle-treated animals. The analysis of variance was performed by one-way ANOVA. A Bonferroni’s significant difference procedure was used as post-hoc comparison. [file 13046_2021_2127_MOESM10_ESM.tiff]
