# Supplementary material for: VEGF-A/VEGFR-1 signalling and chemotherapy-induced neuropathic pain: therapeutic potential of a novel anti-VEGFR-1 monoclonal antibody
Source: J Exp Clin Cancer Res. 2021 Oct 14;40:320. doi: 10.1186/s13046-021-02127-x (PMC8515680; doi:10.1186/s13046-021-02127-x)
Supplement: Supplementary file 8 — Additional file 8: Table S2. Hole Board test. (DOCX 17 kb). The Hole board test was performed 3, 5 and 9 days after the beginning of oxaliplatin treatment (n=5). Each value represents the mean ± SEM. **P<0.01 vs vehicle + vehicle treated animals. The analysis of variance was performed by one-way ANOVA. A Bonferroni’s significant procedure was used as post-hoc comparison. [file 13046_2021_2127_MOESM8_ESM.docx]

**Supplementary Table S2. Hole Board test**

|  | Day 3 | | Day 5 | | Day 9 | |
| --- | --- | --- | --- | --- | --- | --- |
| Treatments | hole | board | hole | board | hole | board |
| vehicle + vehicle | 46.8 ± 10.1 | 71.2 ± 11.1 | 27.4 ± 5.2 | 56.5 ± 6.0 | 21.0 ± 1.0 | 37.0 ± 1.5 |
| vehicle + oxaliplatin | 57.5 ± 4.1 | 66.4 ± 6.6 | 33. 8 ± 7.5 | 44.6 ± 5.8 | 18.0 ± 1.4 | 39.2 ± 4.0 |
| scrambled + oxaliplatin | 49.2 ± 6.0 | 64.3 ± 8.5 | 25.9 ± 6.3 | 47.3 ± 3.8 | 24.4 ± 3.6 | 36.8 ± 2.6 |
| VEGFA-shRNAmir + oxaliplatin | 65.8 ± 4.8 | 174.4 ± 18.3** | 38.5 ± 6.6 | 69.6 ± 6.7 | 22.8 ± 2.7 | 40.4 ± 3.6 |

The Hole board test was performed 3, 5 and 9 days after the beginning of oxaliplatin treatment (n=5). Each value represents the mean ± SEM. **P<0.01 vs vehicle + vehicle treated animals. The analysis of variance was performed by One-way ANOVA. A Bonferroni’s significant procedure was used as post hoc comparison.
